# Safety, accuracy, empathy, reliability, and readability of large language model chatbot responses to public-facing vegetarian and vegan nutrition advice questions: a cross-sectional comparative study

**DOI:** 10.3389/fpubh.2026.1921819

**Published:** 2026-08-03

**Authors:** Jiyong Zhou, Qiongjiao Zhou, Lijun Zhou, Jiajia Wang, Jing Gao, Long Qin, Bing Tan, Ping Fan, Changwei Li

**Affiliations:** 1 Department of Clinical Nutrition, The People’s Hospital of Rongchang District, Chongqing, China; 2 Department of Obstetrics, The Third People’s Hospital of Yongzhou, Yongzhou, China; 3 Department of Hepatobiliary and Hernia Surgery, The Third People’s Hospital of Yongzhou, Yongzhou, China; 4 Department of Clinical Nutrition, Chongqing Yubei District People’s Hospital, Chongqing, China; 5 Department of Clinical Nutrition, The Chenjiaqiao Hospital of Shapingba District of Chongqing, Chongqing, China; 6 Department of Clinical Nutrition, Beibei Affiliated Hospital of Chongqing Medical University, The Ninth People’s Hospital of Chongqing, Chongqing, China; 7 Department of Gastroenterology, The Affiliated Dazu’s Hospital of Chongqing Medical University, Chongqing, China

**Keywords:** accuracy, chatbot health advice, empathy, large language models, readability, reliability, safety, vegetarian and vegan nutrition

## Abstract

**Background:**

Publicly accessible large language model (LLM) chatbots are increasingly used to seek nutrition advice. Although vegetarian and vegan nutrition advice is often perceived as low risk, it may involve supplementation, vulnerable life stages, chronic disease, and symptoms that require clinical assessment.

**Objective:**

To compare the safety, accuracy, empathy, reliability, information quality, and readability of five publicly accessible LLM chatbot products in responding to public-facing vegetarian and vegan nutrition advice questions.

**Methods:**

In this cross-sectional comparative study, 58 predefined public-facing vegetarian and vegan nutrition advice questions were submitted once in English to ChatGPT, Gemini, Microsoft Copilot, DeepSeek, and Doubao through their official web interfaces. The final dataset included 290 chatbot responses. Five blinded raters with clinical nutrition training evaluated safety, accuracy, and empathy using predefined reference-answer anchors, and assessed reliability and information quality using four established instruments: the DISCERN instrument, Ensuring Quality Information for Patients (EQIP), Journal of the American Medical Association (JAMA) benchmark criteria, and Global Quality Score (GQS). Readability was assessed using six formula-based indices: the Automated Readability Index (ARI), Coleman–Liau Index (CL), Flesch–Kincaid Grade Level (FKGL), Gunning Fog Index (GFI), Simple Measure of Gobbledygook (SMOG), and Flesch Reading Ease Score (FRES). Model comparisons were paired by question.

**Results:**

Inter-rater agreement was high for all human-rated outcomes. Potentially harmful responses occurred in all models, although adjusted pairwise safety comparisons were not statistically significant. Recurring harm patterns involved vitamin B12 source reliability, uncontrolled iodine or selenium intake, vulnerable life stages, chronic disease, symptom triage, and poorly traceable or overconfident statements. Accuracy, empathy, reliability, information quality, and readability differed significantly across models. ChatGPT, Copilot, and DeepSeek showed higher accuracy; ChatGPT showed higher empathy; Copilot performed best on DISCERN and EQIP; and Gemini produced the most readable responses.

**Conclusion:**

Publicly accessible LLM chatbots can provide useful general information on vegetarian and vegan nutrition, but their performance is uneven and safety limitations remain. Chatbot-generated advice should be interpreted cautiously, especially for supplementation, vulnerable groups, chronic disease, and symptoms requiring clinical assessment. Future systems should improve safety guidance, referral cues, source traceability, and plain-language communication.

## 1 Introduction

Vegetarian and vegan dietary patterns are increasingly visible in population nutrition and account for a meaningful, although variably estimated, proportion of adults across populations (1–3). This growing visibility has increased public demand for accessible, understandable, and actionable information about vegetarian and vegan nutrition, including diet planning, supplementation, symptom interpretation, and health-related concerns.

Professional guidance generally supports the nutritional adequacy of appropriately planned vegetarian and vegan dietary patterns for adults (4). However, such guidance also emphasizes careful planning, reliable vitamin B12 intake, and attention to vulnerable groups (5). Pediatric guidance is more cautious, stressing growth monitoring, supplementation, and professional supervision for infants, children, and adolescents (6), while evidence on vegan pregnancy remains limited (7). Therefore, public-facing vegetarian and vegan nutrition advice questions may appear simple, but they often require careful distinction between general dietary guidance and situations requiring individualized assessment, supplementation, monitoring, or professional care. This distinction may not be obvious to public users seeking advice outside clinical settings.

Large language model (LLM) chatbots have become readily accessible sources of health and nutrition information. Their conversational format allows public users to receive immediate responses that may appear fluent, personalized, and supportive. Prior work suggests that chatbot responses to patient questions can be perceived as high quality and empathetic (8). However, in health contexts, fluency does not ensure reliability: LLMs may generate inaccurate, incomplete, insufficiently qualified, or weakly sourced advice (9, 10). These concerns are particularly relevant to vegetarian and vegan nutrition advice, where users may act on chatbot-generated recommendations about diet quality, supplementation, symptoms, chronic disease, or vulnerable life stages without further professional review.

Although evidence on chatbot health advice is growing, the conduct and reporting of such studies remain heterogeneous (11), and recent guidance has emphasized transparent reporting of studies evaluating generative AI chatbots for clinical evidence or health advice (12). A 2026 nationally representative U.S. survey of 5,660 adults found that 25% had used an AI tool or chatbot for health information or advice; among those reporting such use in the previous 30 days, 59% had used AI for nutrition or exercise questions (13). However, evidence specific to public-facing vegetarian and vegan nutrition advice remains limited. In particular, it remains unclear how several publicly accessible LLM chatbot products perform in direct comparison when answering the same standardized set of vegetarian and vegan nutrition questions, whether their relative performance differs across safety, accuracy, empathy, reliability, information quality, and readability, and what recurring patterns characterize potentially harmful responses in this domain. This gap is important because questions in this area often combine everyday dietary concerns with issues that may require nutritional adequacy assessment, risk qualification, or referral to health professionals.

To address these gaps, we conducted a cross-sectional comparative study of five publicly accessible LLM chatbot products responding to public-facing vegetarian and vegan nutrition advice questions. The study focused on public users seeking conversational nutrition information and aimed to compare chatbot responses across safety, accuracy, empathy, reliability, information quality, and readability, while also examining qualitative patterns of potentially harmful responses.

## 2 Materials and methods

### 2.1 Study design and reporting

This was a cross-sectional comparative chatbot health advice study evaluating responses generated by publicly accessible large language model (LLM)-based chatbot products to public-facing vegetarian and vegan nutrition advice questions. Prespecified questions were submitted to five generative AI-driven chatbot products through their official web-based interfaces, and the outputs were evaluated against predefined reference-answer anchors. The unit of comparison was the user-facing chatbot product under its recorded interface configuration, rather than the underlying base model alone.

The study was designed and reported in accordance with the Chatbot Assessment Reporting Tool (CHART) statement (12). Model identifiers, access routes, query dates and location, prompt sources, response-collection procedures, reference standards, evaluation procedures, sample-size determination, statistical methods, ethics and privacy considerations, and data availability are reported in the manuscript or Supplementary materials. The completed CHART checklist is provided as Supplementary Table S1.

### 2.2 Question-set and prompt development

A structured question set was developed to reflect public-facing nutrition questions about vegetarian and vegan dietary patterns, including questions from people considering, initiating, maintaining, or caring for someone following vegetarian or vegan diets. JZ and CL generated an initial pool of 192 candidate questions from multiple public information-seeking sources, including Google Trends^1^, online health information websites and forums^2^^,^^3^^,^^4^, and vegetarian- or vegan-related websites^5^. Sources in both Chinese and English were used to identify recurring public question themes, and the final prompts were drafted in English for standardized chatbot querying. Details of the question pool, screening decisions, and final question selection are provided in Supplementary File S1.

JZ and QZ screened the 192 candidate questions for eligibility. Questions were eligible if they were public-facing, nutrition-related, relevant to vegetarian or vegan dietary patterns, and answerable using general evidence-based nutrition guidance. Duplicate, unclear, non-nutrition-related, purely opinion-based, and highly individualized diagnostic or treatment questions were excluded. Disagreements were resolved through consultation with CL, who made the final decision when consensus could not be reached. The final question set included 58 questions covering general diet quality, protein and energy intake, micronutrients and supplementation, vulnerable life stages, chronic disease, and symptom-related concerns.

For chatbot querying, each final prompt consisted only of the question text. No instructions were added regarding response length, tone, citation format, safety advice, or answer structure, so that the queries would approximate ordinary public use of LLM chatbots. Before querying, the wording of the final questions was reviewed by 10 lay public representatives to improve readability, clarity, and natural expression from a non-specialist perspective. These contributors were community-dwelling adults with no professional background in medicine, nursing, nutrition, pharmacy, or artificial intelligence. Their involvement was limited to wording refinement. They did not participate in evidence mapping, chatbot querying, response evaluation, statistical analysis, or interpretation of findings.

### 2.3 Reference standard and question–evidence mapping

A predefined reference standard was established before model-response evaluation. For each of the 58 questions, JZ and LZ developed reference-answer anchors specifying the expected elements of a safe, accurate, and complete public-facing nutrition response. These anchors covered key nutrients, practical dietary strategies, supplementation considerations, clinical red flags, and circumstances requiring professional assessment. They were framed for public nutrition consultation and were not intended to replace individualized clinical diagnosis, laboratory testing, or treatment.

JZ and LZ mapped each question to relevant guideline, consensus, position-paper, systematic-review, specialty-review, and disease-specific clinical or professional evidence. The evidence base covered adult vegetarian and vegan dietary patterns, practical vegan nutrition, plant-based diet safety, pediatric and adolescent vegan diets, pregnancy, complementary feeding, chronic kidney disease, gout/hyperuricemia, thyroid disease and iodine safety, sports nutrition, plant-based meat alternatives, and food allergy considerations (4–7, 14–27). Disagreements during reference-standard development or evidence mapping were resolved through consultation with CL, who made the final decision when consensus could not be reached. The complete question–evidence mapping table, source abbreviations, reference-answer anchors, and corresponding evidence sources are provided in Supplementary File S2.

### 2.4 Chatbot selection, model identifiers, and access conditions

The evaluated chatbot products were ChatGPT, Gemini, Microsoft Copilot, DeepSeek, and Doubao. They were purposively selected to capture heterogeneity across developers and product ecosystems, including internationally prominent platforms and high-visibility China-developed platforms. All selected products were general-purpose chatbot systems that were publicly accessible through official web interfaces and available during the study period. The study was designed as a focused comparative evaluation of this deliberately selected set rather than as an exhaustive benchmark of all chatbot products available at the time; other systems were therefore outside the predefined scope of the study. All systems were accessed through their official web-based interfaces; no API access, local deployment, custom fine-tuning, investigator-developed retrieval-augmented generation, or additional model deployment was used.

The recorded user-facing identifiers and configurations were ChatGPT (GPT-5.4 Thinking; Standard mode), Gemini (Gemini web interface; Gemini 3.5 Flash; Think mode enabled), Microsoft Copilot (Copilot web interface; Think Deeper mode), DeepSeek (DeepSeek-V4-Pro; Expert mode, Deep Thinking, and Intelligent Search enabled), and Doubao (Seed-Doubao V3.7.2; Thinking mode enabled). Backend routing, training data, system prompts, exact underlying model identifiers, and complete deployment parameters were not always disclosed for all products. Full details of the recorded user-facing identifiers and configurations are provided in Supplementary Table S2.

### 2.5 Query strategy and response collection

Queries were conducted from 20 May 2026 to 27 May 2026 in Chongqing, China. Each of the 58 questions was submitted once to each chatbot in English. This single-response protocol was chosen to approximate the response that a public user might receive during a single first-contact interaction, rather than to assess reproducibility across repeated queries. No additional role instruction, individualized clinical information, supplementary context, or follow-up prompt was provided.

JZ performed the querying procedure, and CL supervised response collection and organization of the analytic response record. To reduce contextual carryover, each prompt was submitted in a single-turn, separate-session format. After each response was saved, the corresponding prompt-response pair was deleted from the visible conversation history, and a new chat session was created before the next question was submitted. History-based memory or personalization was disabled where available. When the interface provided multiple modes or model options, the public-facing mode or configuration recorded in Supplementary Table S2 was used.

All outputs were saved verbatim immediately after generation. Non-answer interface elements, advertisements, suggested follow-up questions, and unrelated system content were removed from the analytic plain-text dataset. Substantive answer text was retained without rewriting. The complete prompt set and verbatim plain-text responses are provided in Supplementary File S3.

### 2.6 Outcome measures and scoring instruments

The unit of human evaluation was the question–model response. The human-rated outcome domains were safety, accuracy, empathy, reliability, and information quality. Safety was coded as safe or potentially unsafe, with response-level classification determined by majority rating across the five blinded raters. Potentially unsafe responses were defined as responses that could plausibly mislead public users, omit important safety warnings, encourage inappropriate supplementation or self-treatment, delay clinical evaluation, underemphasize red-flag symptoms, or provide overly reassuring guidance for vulnerable groups or chronic disease contexts.

Accuracy and empathy were rated on 1–5 scales. Accuracy was assessed according to consistency with the corresponding reference-answer anchor, and empathy was assessed according to the quality of supportive, respectful, nonjudgmental, and patient-centered communication. Reliability and information quality were assessed using established instruments: DISCERN for reliability and quality of written consumer health information (28), EQIP for the quality of written health information (29), JAMA for authorship, attribution, disclosure, and currency of online medical information (30), and the GQS as an overall measure of information quality and usefulness (31). For accuracy, empathy, DISCERN, EQIP, JAMA, and GQS, response-level scores were calculated as the mean of the five independent rater scores. Detailed scoring criteria, rater training procedures, rating instructions, and instrument definitions are provided in Supplementary Table S3.

### 2.7 Human rating process and outcomes

Five raters, JW, JG, LQ, BT, and PF, including two nutrition physicians and three nutrition technicians, independently evaluated all model responses. All raters had clinical nutrition training, at least 2 years of relevant professional experience, and professional titles at junior level or above. The raters were not involved in study design, model querying, question selection, reference-answer anchor development, or statistical analysis.

Before formal scoring, the raters received standardized training on the study objectives, reference-answer anchors, scoring instruments, rating criteria, and examples of potentially unsafe responses. Formal scoring began only after raters were familiar with the scoring rules. Responses were anonymized and presented with coded identifiers; model names and chatbot-specific identifiers were removed before rating. Raters evaluated responses independently using the same structured scoring sheet. Disagreements were not resolved by consensus for the primary analysis. Rater-level scores were retained for inter-rater agreement analysis. No patients or public contributors were involved in response evaluation.

### 2.8 Sample size

The analytic sample was derived from the prespecified question-development process. From an initial pool of 192 candidate questions, 58 public-facing vegetarian and vegan nutrition questions were retained for model evaluation. Each question was submitted once to each of the five chatbot products, yielding 290 model responses for analysis.

All 290 responses were included in the human-rated evaluation of safety, accuracy, empathy, reliability, and information quality, and the same complete English-language answer texts were used for readability assessment. Because five independent raters evaluated each response, the human-rating dataset contained 1,450 response–rater evaluations before aggregation of rater scores. No formal sample-size calculation was performed because this was a cross-sectional comparative evaluation of a predefined public-facing question set rather than a hypothesis-driven clinical trial.

### 2.9 Qualitative analysis of potentially harmful responses

Responses classified as potentially unsafe were further reviewed descriptively after completion of the primary safety classification. JW and JG conducted this review at both the question and response levels. Potential harm patterns were grouped using an operational risk taxonomy covering B12 source reliability and neurologic red flags, mineral toxicity and uncontrolled dosing, high-risk life-stage advice, chronic disease and symptom triage, and traceability, overconfidence, and applicability concerns. This descriptive qualitative analysis did not alter the primary quantitative safety classification. The full taxonomy, operational definitions, and detailed analysis of safety-flagged responses are provided in Supplementary File S4.

### 2.10 Readability assessment

Readability was assessed for all 290 complete English-language chatbot responses. Redundant whitespace and non-answer interface content were removed, but substantive answer text was not rewritten or simplified. Six formula-based readability indices were calculated: Flesch Reading Ease Score (FRES), Flesch–Kincaid Grade Level (FKGL), Simple Measure of Gobbledygook (SMOG), Automated Readability Index (ARI), Coleman–Liau Index (CL), and Gunning Fog Index (GFI) (32–37). The six indices were selected because they rely on different textual features and provide complementary estimates of reading ease and grade-level difficulty. Using multiple formulas reduced dependence on any single algorithm and allowed assessment of whether between-product readability patterns were consistent across measures. Higher FRES indicates easier readability, whereas higher values for the other five indices indicate greater reading difficulty. The formulas, required text variables, and interpretation of each readability metric are provided in Supplementary Table S4.

### 2.11 Statistical analysis

All analyses were performed using R version 4.5.1. The analytic dataset was organized in long format with question, model, metric, score, and rater-level columns where applicable. Because the same 58 questions were answered by all five chatbot products, model comparisons were treated as paired by question.

Safety was coded as 0 for safe and 1 for potentially unsafe or potentially harmful. Safety outcomes were summarized as counts and percentages. Inter-rater agreement for safety was assessed using Fleiss’ *kappa* (38). For non-binary human-rated outcomes, inter-rater agreement was assessed using two-way random-effects, absolute-agreement, single-measure intraclass correlation coefficients, ICC(2,1) (39). Response-level scores were calculated by averaging the five independent rater scores for each question–model response.

Overall safety differences among models were assessed using Cochran’s *Q* test (40), and pairwise safety comparisons used McNemar tests. Paired safe-rate differences, matched odds ratios, and bootstrap 95% confidence intervals were reported.

For non-binary outcomes, descriptive statistics were reported as both mean ± standard deviation and median with interquartile range. Mean ± standard deviation was used to describe average score levels and facilitate comparison with prior quantitative evaluations, whereas median with interquartile range was reported because several outcomes were ordinal, bounded, or non-normally distributed. Overall model differences for non-binary outcomes were assessed using Friedman tests, with Kendall’s *W* reported as the effect size. Pairwise comparisons used paired Wilcoxon signed-rank tests. Paired mean differences, paired median differences, paired rank-biserial correlations, and bootstrap 95% confidence intervals were reported where applicable.

Pairwise *p* values were adjusted using the Benjamini–Hochberg method to control the false discovery rate (41). All tests were two-sided, and adjusted *p* < 0.05 was considered statistically significant. Readability indices were analyzed using the same paired-by-question framework. Figures were generated using ggplot2 and patchwork.

### 2.12 Ethics and privacy

This study analyzed publicly generated chatbot responses and did not involve patients, clinical interventions, medical records, identifiable personal information, or individual-level health data. Lay public representatives contributed only to question wording refinement and did not provide personal health information or participate in response evaluation. No personal user data, account-level identifiers, usernames, individual forum posts, or identifiable health records were collected. Therefore, ethics approval was not required.

## 3 Results

### 3.1 Study flow and response dataset

**Figure 1:**
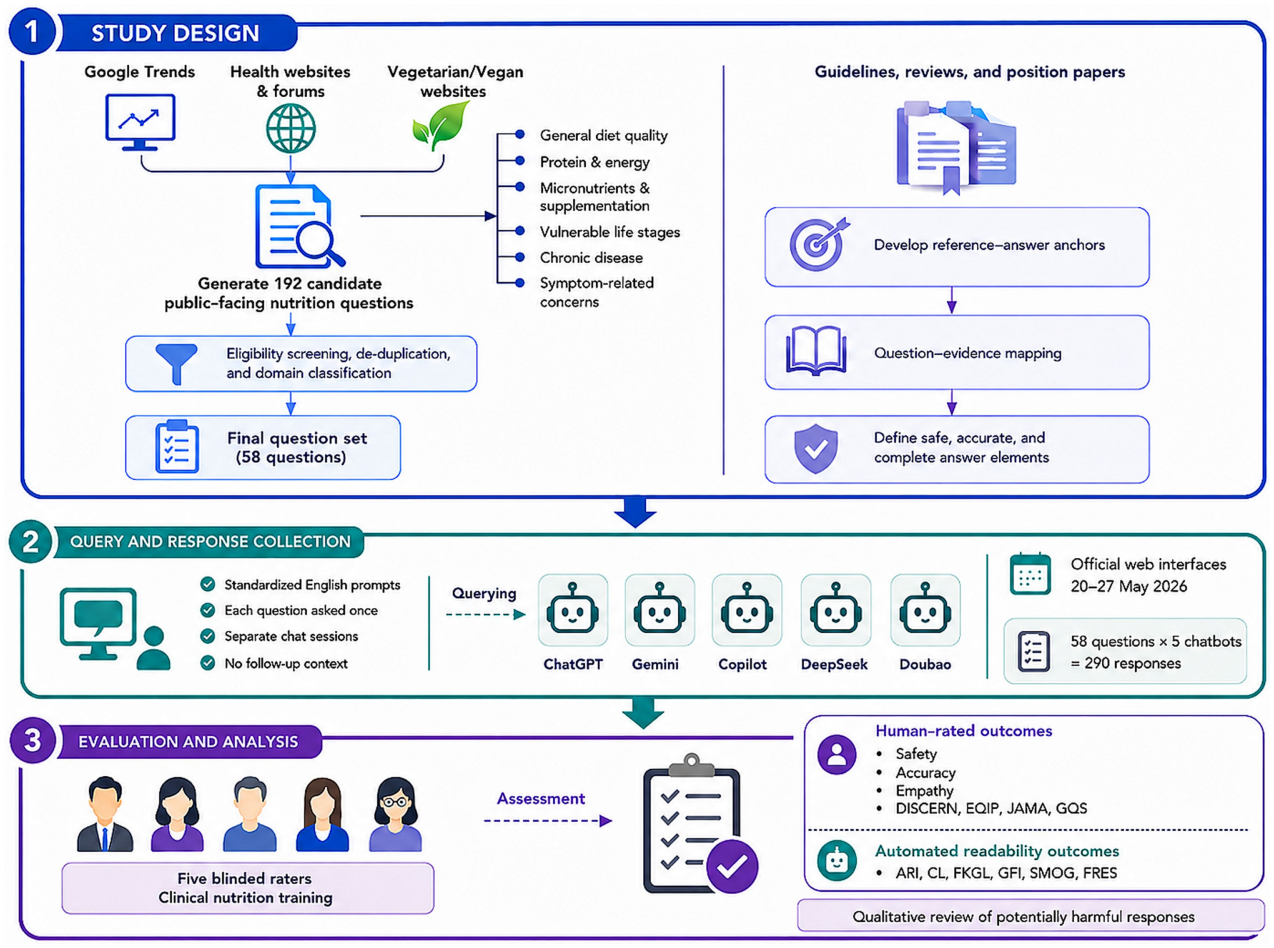
Study workflow and CHART-guided evaluation structure. The figure summarizes question identification, screening, reference-answer anchor development, chatbot querying, response collection, and outcome evaluation. From 192 candidate questions, 58 public-facing vegetarian and vegan nutrition advice questions were retained and submitted once to each of five publicly accessible LLM chatbot products, yielding 290 chatbot responses. Responses were evaluated using human-rated measures, automated readability indices, and qualitative review of potentially harmful responses. ARI, Automated Readability Index; CHART, Chatbot Assessment Reporting Tool; CL, Coleman–Liau Index; DISCERN, DISCERN instrument; EQIP, Ensuring Quality Information for Patients; FKGL, Flesch–Kincaid Grade Level; FRES, Flesch Reading Ease Score; GFI, Gunning Fog Index; GQS, Global Quality Score; JAMA, Journal of the American Medical Association; LLM, large language model; SMOG, Simple Measure of Gobbledygook.

Figure 1 summarizes the study workflow. After question identification, de-duplication, eligibility screening, wording refinement, and domain classification, 58 public-facing vegetarian and vegan nutrition advice questions were retained for evaluation. Each question was submitted once to each of the five LLMs, yielding 290 complete responses. All 290 responses were included in the assessment of safety, accuracy, empathy, reliability, information quality, and readability.

### 3.2 Inter-rater agreement

Inter-rater agreement estimates are presented in Supplementary Table S5. For safety ratings, the Fleiss’ kappa was 0.823. The ICC(2,1) values were 0.875 for accuracy, 0.882 for empathy, 0.877 for DISCERN, 0.878 for EQIP, 0.872 for JAMA, and 0.876 for GQS, with corresponding statistical test results reported in Supplementary Table S5.

### 3.3 Safety, accuracy, and empathy

**Figure 2:**
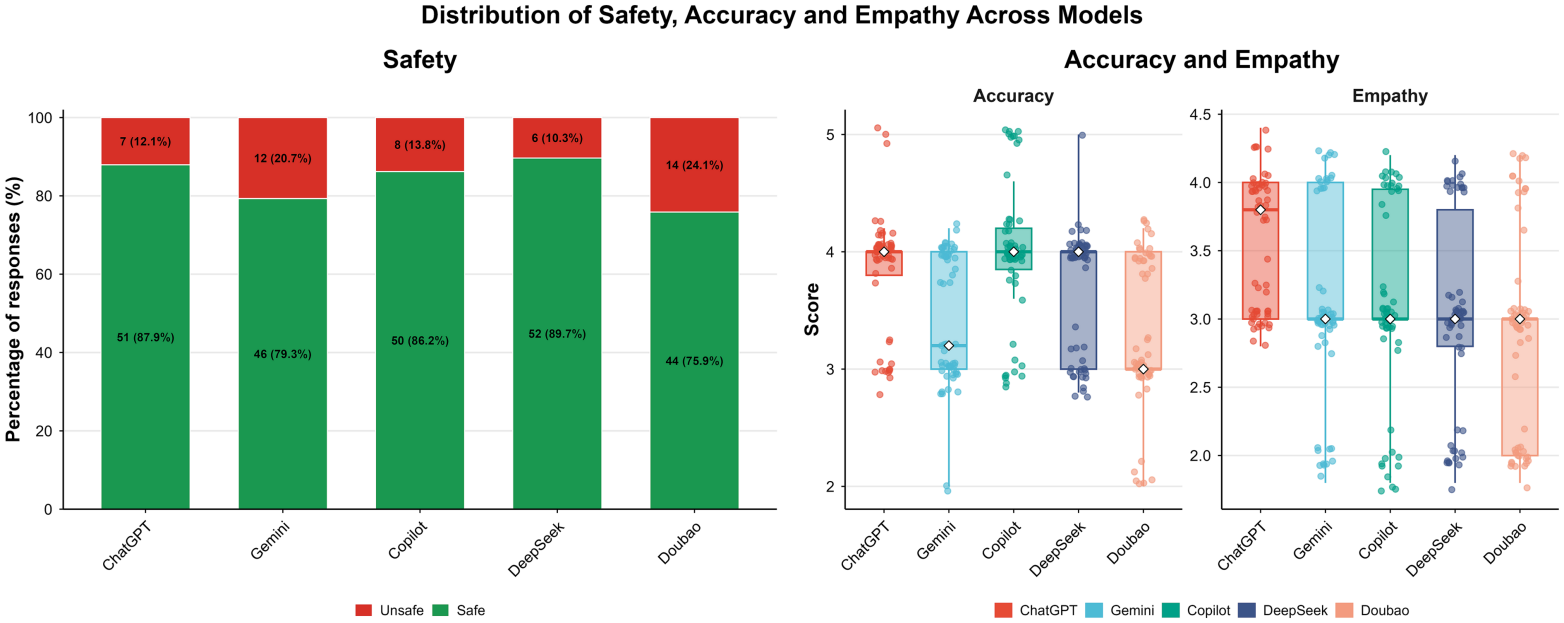
Distribution of safety, accuracy, and empathy across chatbot products. Safety was classified as safe or potentially unsafe by majority judgment across five blinded raters. Accuracy and empathy were rated on 1–5 scales, with higher scores indicating better agreement with reference-answer anchors and stronger patient-centered communication, respectively. Boxplots show medians and interquartile ranges; points represent individual chatbot responses, and white diamonds indicate mean scores.

Safety, accuracy, and empathy outcomes are presented in Table 1 and Figure 2. Safe-response rates were 89.7% for DeepSeek; 87.9% for ChatGPT; 86.2% for Copilot; 79.3% for Gemini; and 75.9% for Doubao. Pairwise McNemar tests with Benjamini–Hochberg correction did not identify statistically significant differences in safety between models. Detailed safety comparisons are provided in Supplementary Table S6.

**Table 1:** Accuracy and empathy scores of chatbot responses to public-facing vegetarian and vegan nutrition advice questions by model.

Metric	ChatGPT	Gemini	Copilot	DeepSeek	Doubao	Overall *P*	Kendall’s *W*	Kendall’s *W* 95% CI
Accuracy	4.00 [3.80, 4.00]	3.20 [3.00, 4.00]	4.00 [3.85, 4.20]	4.00 [3.00, 4.00]	3.00 [3.00, 4.00]	<0.001	0.220	0.150–0.326
Empathy	3.80 [3.00, 4.00]	3.00 [3.00, 4.00]	3.00 [3.00, 3.95]	3.00 [2.80, 3.80]	3.00 [2.00, 3.00]	<0.001	0.112	0.062–0.208

Values are median [Q1, Q3]. Overall *P*-values were obtained using Friedman tests.

A qualitative review of safety-flagged responses identified 47 potentially harmful responses across 14 questions. These responses clustered around five recurring risk domains: vitamin B12 source reliability and neurologic red flags, mineral toxicity and uncontrolled dosing, high-risk life-stage advice, chronic disease and symptom triage, and traceability or overconfidence concerns. Safety-flagged questions included items on B12 sources, seaweed iodine, Brazil nut selenium, pregnancy, infant feeding, adolescent and older-adult nutrition, chronic kidney disease, thyroid disease, persistent fatigue, and neurologic symptoms after adopting a vegan diet. The number of safety-flagged responses was 14 for Doubao, 12 for Gemini, 8 for Copilot, 7 for ChatGPT, and 6 for DeepSeek. The question-level and response-level qualitative safety analyses are provided in Supplementary File S4.

Accuracy differed significantly across models (*p* < 0.001; Kendall’s *W* = 0.220, 95% CI 0.150–0.326). Median accuracy scores were highest for ChatGPT, Copilot, and DeepSeek, whereas Gemini and Doubao showed lower median scores. Empathy also differed significantly across models (*p* < 0.001; Kendall’s *W* = 0.112, 95% CI 0.062–0.208), with ChatGPT showing the highest median empathy score. *Post hoc* comparisons for accuracy and empathy are provided in Supplementary Table S7.

### 3.4 Reliability and quality of information

**Figure 3:**
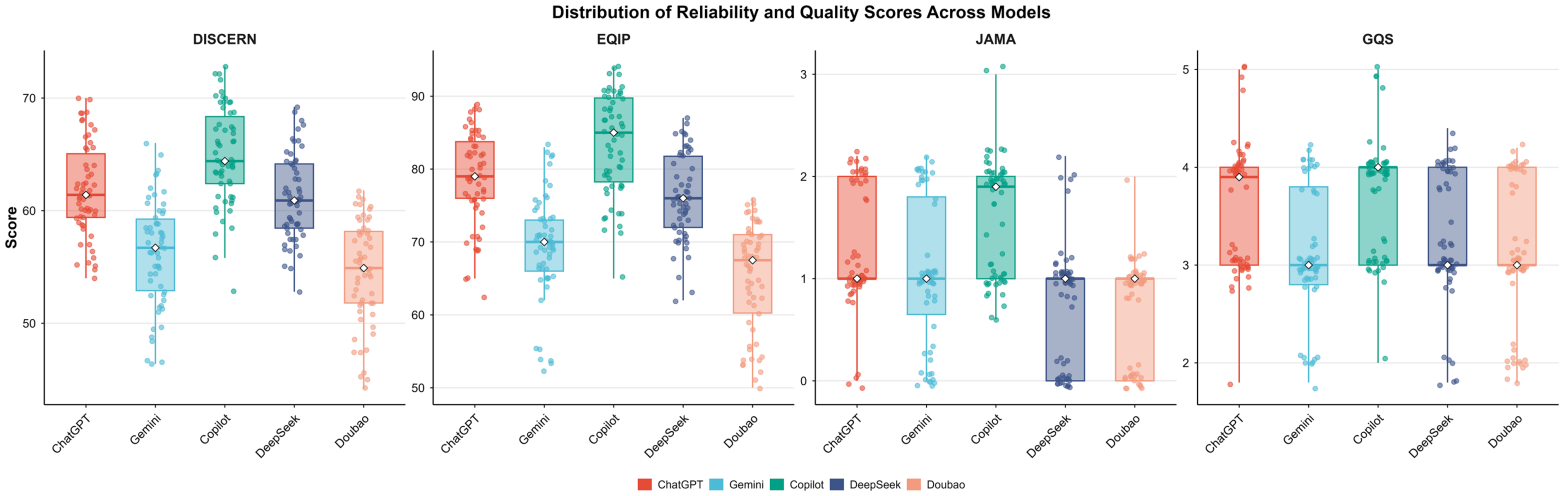
Distribution of reliability and information-quality scores across chatbot products. Responses were evaluated using DISCERN, EQIP, JAMA, and GQS. Higher scores indicate better reliability, information quality, transparency, or overall usefulness according to the corresponding instrument. Boxplots show medians and interquartile ranges; points represent individual chatbot responses, and white diamonds indicate mean scores.

Reliability and information-quality outcomes are presented in Table 2 and Figure 3. Significant overall differences were observed across models for DISCERN, EQIP, JAMA, and GQS. Copilot generally achieved the highest reliability and information-quality scores, particularly for DISCERN and EQIP, while Doubao generally showed the lowest scores. The largest model differences were observed for EQIP (Kendall’s *W* = 0.696, 95% CI 0.628–0.769) and DISCERN (Kendall’s *W* = 0.607, 95% CI 0.545–0.677). Significant pairwise *post hoc* comparisons are reported in Supplementary Table S8.

**Table 2:** Reliability and information-quality scores of chatbot responses to public-facing vegetarian and vegan nutrition advice questions by model.

Metric	ChatGPT	Gemini	Copilot	DeepSeek	Doubao	Overall *P*	Kendall’s *W*	Kendall’s *W* 95% CI
DISCERN	61.40 [59.40, 65.05]	56.70 [52.90, 59.25]	64.40 [62.40, 68.35]	60.90 [58.45, 64.15]	54.90 [51.80, 58.15]	<0.001	0.607	0.545–0.677
EQIP	79.00 [76.00, 83.75]	70.00 [66.00, 73.00]	85.00 [78.25, 89.75]	76.00 [72.00, 81.75]	67.50 [60.25, 71.00]	<0.001	0.696	0.628–0.769
JAMA	1.00 [1.00, 2.00]	1.00 [0.65, 1.80]	1.90 [1.00, 2.00]	1.00 [0.00, 1.00]	1.00 [0.00, 1.00]	<0.001	0.223	0.148–0.332
GQS	3.90 [3.00, 4.00]	3.00 [2.80, 3.80]	4.00 [3.00, 4.00]	3.00 [3.00, 4.00]	3.00 [3.00, 4.00]	<0.001	0.150	0.084–0.262

Values are median [Q1, Q3]. Overall *P*-values were obtained using Friedman tests.

### 3.5 Readability

**Figure 4:**
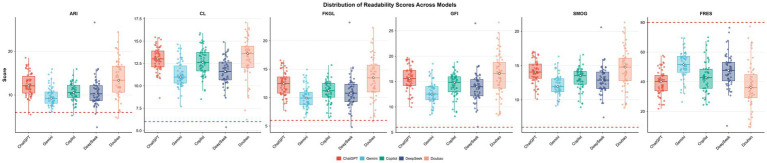
Distribution of readability scores across chatbot products. Readability was assessed using ARI, CL, FKGL, GFI, SMOG, and FRES. For ARI, CL, FKGL, GFI, and SMOG, higher scores indicate greater reading difficulty; for FRES, higher scores indicate easier readability. Boxplots show score distributions by chatbot product; points represent individual chatbot responses, and white diamonds indicate mean scores.

Readability results are summarized in Table 3 and Figure 4. Significant overall differences were found for all six readability metrics, including ARI, CL, FKGL, GFI, SMOG, and FRES (all *p* < 0.001), with Kendall’s *W* values ranging from 0.275 for SMOG (95% CI 0.181–0.403) to 0.385 for FRES (95% CI 0.267–0.522). For grade-level or difficulty indices, lower values indicate easier readability; Gemini generally had the lowest difficulty scores, whereas Doubao and ChatGPT generally showed greater reading difficulty. For FRES, where higher scores indicate easier readability, Gemini had the highest mean score, followed by DeepSeek and Copilot, while Doubao and ChatGPT had lower scores. Significant pairwise readability comparisons are provided in Supplementary Table S9.

**Table 3:** Readability scores of chatbot responses to public-facing vegetarian and vegan nutrition advice questions by model.

Metric	ChatGPT	Gemini	Copilot	DeepSeek	Doubao	Overall *P*	Kendall’s *W*	Kendall’s *W* 95% CI
ARI	12.47 ± 2.59	9.66 ± 2.19	10.67 ± 2.03	10.66 ± 3.31	13.56 ± 4.48	<0.001	0.292	0.193–0.425
CL	12.99 ± 1.31	11.25 ± 1.42	12.67 ± 1.57	11.57 ± 1.60	13.16 ± 2.28	<0.001	0.356	0.236–0.506
FKGL	12.47 ± 2.10	9.99 ± 1.87	11.07 ± 1.91	10.95 ± 2.68	13.28 ± 3.70	<0.001	0.326	0.228–0.456
GFI	15.66 ± 2.35	12.82 ± 2.20	14.36 ± 2.27	13.92 ± 2.87	16.32 ± 4.14	<0.001	0.298	0.197–0.430
SMOG	14.12 ± 1.66	12.16 ± 1.57	13.13 ± 1.55	12.91 ± 1.95	14.54 ± 2.89	<0.001	0.275	0.181–0.403
FRES	39.51 ± 8.40	51.36 ± 9.23	43.03 ± 10.37	47.49 ± 10.93	37.26 ± 15.12	<0.001	0.385	0.267–0.522

Values are mean ± SD. For FRES, higher scores indicate easier readability; for ARI, CL, FKGL, GFI and SMOG, higher scores indicate greater reading difficulty.

## 4 Discussion

In this cross-sectional comparative study, five publicly accessible LLM-based chatbot products generated 290 responses to 58 public-facing vegetarian and vegan nutrition advice questions. These systems provided broadly useful nutrition information, but their performance was uneven across evaluation dimensions. Potentially harmful content was identified in all five chatbot products, although adjusted pairwise safety comparisons were not statistically significant. Accuracy, empathy, reliability, information quality, and readability differed significantly across products, indicating that chatbot performance in public-facing nutrition advice should not be reduced to a single overall score.

These findings are clinically relevant because vegetarian and vegan nutrition is often perceived as low risk but can involve high-consequence advice. Professional guidance supports appropriately planned vegetarian and vegan diets but also emphasizes nutrient adequacy, supplementation where needed, and caution for vulnerable groups and specific nutrients (4, 6, 7, 42, 43). Therefore, the key issue is not whether chatbots can answer general diet questions, but whether they can consistently recognize when ordinary nutrition advice becomes clinical safety advice.

Safety concerns occurred across the evaluated chatbot products. Descriptive safe-response rates varied, but pairwise safety differences were not statistically significant after correction for multiple testing. More informative than ranking the products was the pattern of safety-flagged responses. The qualitative safety analysis identified 47 potentially harmful responses across 14 questions, mainly involving unreliable vitamin B12 source advice, neurologic red flags, mineral toxicity and uncontrolled dosing, vulnerable life stages, chronic disease, symptom triage, and traceability or overconfidence concerns. Similar concerns have been reported in emergency-care chatbot evaluation, where responses sometimes omitted urgent-care advice, provided incomplete information, or lacked reliable sources (44). Broader clinical discussions of LLM use also emphasize hallucinations, overconfident outputs, and incomplete contextual reasoning as persistent risks in medical applications (45).

In the present study, many safety-flagged responses were not entirely incorrect; rather, they combined correct general information with unsafe ambiguity. This was most apparent for questions about vitamin B12, seaweed iodine, Brazil nuts as a selenium source, pregnancy, infant feeding, chronic kidney disease, thyroid disease, fatigue, and neurologic symptoms after adopting a vegan diet. For public users, such ambiguity may be difficult to detect. Briefly, the relevant evidence supports reliable vitamin B12 supplementation or fortified foods (46, 47), cautious iodine advice because of insufficiency, excess, and seaweed variability (48–50), and cautious wording around Brazil nuts because selenium content and exposure may vary (51, 52). These findings suggest that public-facing chatbots need stricter safety templates for nutrient-source questions: state the core recommendation first, avoid ambiguous exceptions, discourage uncontrolled supplementation, and identify when clinical or dietetic assessment is needed.

Accuracy differed significantly across chatbot products. ChatGPT, Copilot, and DeepSeek showed higher median accuracy scores than Gemini and Doubao, suggesting closer alignment with predefined reference-answer anchors for questions requiring nutrient prioritization, reliable supplementation, red-flag recognition, and referral advice. This is consistent with dietitian-based evaluation showing that LLM-generated dietary guidance can vary in nutrition literacy, completeness, and practical suitability (53). However, accuracy alone did not eliminate safety concerns. A response may be broadly accurate but still unsafe if it omits urgency for numbness or tingling, provides supplement doses without clinical context, or reassures a high-risk user without emphasizing monitoring. Future evaluations should therefore keep safety and accuracy as separate outcomes rather than treating safety as a subset of factual correctness.

Empathy also differed across chatbot products, with ChatGPT showing relatively higher median empathy scores. This is relevant because prior work has reported that chatbot responses to patient questions may be perceived as more empathetic than physician responses in some online settings (8). However, empathy should not be interpreted as clinical adequacy. A reassuring tone may improve acceptability but may also reduce perceived urgency if warning signs are not clearly communicated. For nutrition-related chatbot advice, patient-centered language should acknowledge the user’s concern, provide practical next steps, and state when self-management is insufficient.

Reliability and information-quality metrics also differed across products. Copilot generally achieved higher DISCERN and EQIP scores, whereas Doubao generally showed lower scores. These differences suggest that performance varied not only in factual accuracy but also in response structure, completeness, balance, and usability. Evaluation of LLMs on registered dietitian examination tasks also suggests that performance may vary by nutrition domain, question difficulty, and prompting strategy (54). In vegetarian and vegan nutrition, high-quality advice should specify nutrients requiring attention, reliable sources, when supplementation may be needed, and how advice changes in pregnancy, infancy, older age, chronic kidney disease, thyroid disease, or symptoms suggestive of deficiency.

JAMA scores were generally limited, indicating a persistent transparency problem in chatbot-generated health information. General-purpose chatbots often provide advice without consistently displaying source dates, authorship, or the evidentiary basis for specific claims. This matters in nutrition because recommendations may depend on national fortification practices, supplement availability, local dietary patterns, laboratory values, pregnancy and pediatric guidance, and disease-specific context. The findings therefore support better source traceability and clearer distinction between general educational advice and individualized clinical guidance.

The present study also illustrates the value of CHART-aligned reporting. Prior evidence has shown substantial heterogeneity in the conduct and reporting of chatbot health advice studies (11). Because public chatbot products change over time, transparent reporting remains essential for interpretation. Accordingly, the present findings should be understood as product-level comparisons under recorded web-interface configurations, rather than fixed properties of the underlying base models.

Readability differed significantly across all six indices. Gemini generally produced easier-to-read responses, whereas Doubao and ChatGPT tended to generate more difficult text on grade-level metrics. These results suggest that better performance in accuracy, empathy, or information quality does not necessarily translate into easier public communication. This issue is important because limited health literacy is associated with differences in health knowledge, health behavior, and access to appropriate care (55). Public users may turn to chatbots expecting simpler explanations than those provided by guidelines, scientific articles, or clinical encounters. However, readability should not be improved by removing essential safety information. AI-assisted patient education may improve readability, but easier wording does not automatically ensure recommended reading levels or preservation of clinical meaning (56). Future systems should therefore optimize safe readability rather than simplicity alone. Public-facing nutrition responses may benefit from a direct answer, a few key action points, brief safety warnings for high-risk users, and clear advice to seek professional care when appropriate.

This study has several strengths. It used a structured question-development process and a predefined set of 58 public-facing vegetarian and vegan nutrition advice questions covering diet quality, protein and energy intake, micronutrients, supplementation, vulnerable life stages, chronic disease, and symptom-related concerns. Responses were evaluated against reference-answer anchors developed from guideline, consensus, position-paper, systematic-review, and specialty-review sources. Five blinded raters with clinical nutrition training independently evaluated all responses, and agreement estimates supported the consistency of human-rated outcomes. The study assessed multiple complementary dimensions, including safety, accuracy, empathy, DISCERN, EQIP, JAMA, GQS, and six readability indices, and combined quantitative scoring with qualitative safety analysis to identify harm patterns that aggregate scores alone may not capture.

Several limitations should be considered. First, publicly accessible chatbot products are dynamic, and their outputs may be influenced by user-facing modes, retrieval functions, reasoning settings, backend routing, system prompts, safety layers, and deployment updates that are not fully visible to investigators. Therefore, the findings should be interpreted as product-level comparisons under the recorded query settings, dates, and location, not as definitive comparisons of underlying base LLMs or future versions. Second, each prompt was submitted once to each chatbot. Although this protocol was intended to approximate the response that a public user might receive during a single first-contact interaction, it did not assess response reproducibility, temporal stability, or within-product response variability. The findings therefore represent the sampled responses generated under the recorded conditions rather than the full range of outputs that might occur across repeated queries or over time. Future studies should incorporate repeated querying and assessments at multiple time points to quantify within-product variability and temporal stability. Third, questions were submitted in English and without individualized clinical information or follow-up interaction; results may differ in other languages, cultural contexts, regions, dietary patterns, user profiles, repeated queries, or multi-turn conversations. Fourth, although raters were blinded and agreement estimates were consistent, human scoring remains partly subjective, especially for empathy and information quality. Fifth, the reference-answer anchors were based on evidence mapping and expert judgment rather than a single universal gold standard; for disease-specific questions, these anchors should be interpreted as public-facing nutrition advice standards supported by general nutrition sources and disease-specific guidance, rather than as comprehensive clinical management guidelines. Sixth, readability formulas estimate textual difficulty but do not directly measure comprehension, numeracy, trust, cultural appropriateness, or behavior after reading. Finally, the study evaluated static chatbot outputs rather than real-world interactions in which users may disclose additional symptoms, ask follow-up questions, or act on advice without verification.

For the public, chatbot responses to public-facing vegetarian and vegan nutrition advice questions should be treated as educational starting points rather than substitutes for individualized medical or dietetic assessment. Caution is especially warranted for questions involving pregnancy, infants, children, adolescents, older adults, chronic kidney disease, thyroid disease, persistent fatigue, neurologic symptoms, or supplement dosing. For clinicians and dietitians, chatbot outputs may help identify common public concerns and misconceptions, but they should be reviewed before being incorporated into patient education, particularly in high-risk scenarios.

For developers, the findings indicate several priorities for public-facing chatbot products. Systems should provide consistent vitamin B12 guidance, avoid ambiguous exceptions for unreliable B12 sources, use cautious language for iodine and selenium sources, discourage uncontrolled supplementation, and trigger referral-oriented responses for vulnerable life stages, chronic disease, and neurologic red flags. They should also improve source traceability, date awareness, and plain-language communication. At the product level, future evaluations should distinguish, where possible, the contribution of base models, retrieval functions, reasoning modes, interface settings, and safety layers to observed response quality.

For researchers, future studies should assess response reproducibility, multilingual performance, real-user comprehension, prompt strategies, retrieval-augmented or guideline-grounded systems, and clinical or behavioral outcomes. Recent sports-nutrition evaluations of LLM chatbots suggest that performance can vary by question type and that professional review remains important when advice may affect training, supplementation, or health-related decisions (57). Studies should continue to report model identifiers, access routes, query settings, complete outputs, reference standards, and evaluation procedures in accordance with CHART. Such reporting is important because chatbot health advice studies are time-sensitive, and transparent methods are needed for readers to judge whether findings remain applicable after model updates.

## 5 Conclusion

Publicly accessible LLM chatbot products can provide useful general information in response to public-facing vegetarian and vegan nutrition advice questions, but their performance remains uneven across evaluation dimensions and safety limitations persist. Chatbot-generated advice should be interpreted cautiously, particularly for questions involving supplementation, vulnerable groups, chronic disease, or symptoms requiring clinical assessment. Future chatbot development should prioritize consistent safety guidance for high-risk nutrition scenarios, explicit referral cues for red-flag symptoms, improved source traceability, and plain-language communication that preserves clinically important cautions.

## Data Availability

The original contributions presented in the study are included in the article/Supplementary material, further inquiries can be directed to the corresponding author.

## Footnotes

1
https://trends.google.com

2
https://dxy.com

3
https://tieba.baidu.com

4
https://www.reddit.com

5
https://vegsoc.org

## Supplementary material

SUPPLEMENTARY FILE S1 Question pool development, screening decisions, and final question selection.

SUPPLEMENTARY FILE S2 Question–evidence mapping table and reference-answer anchors.

SUPPLEMENTARY FILE S3 Complete plain-text chatbot responses.

SUPPLEMENTARY FILE S4 Qualitative analysis of potentially harmful responses.

SUPPLEMENTARY FILE S5 Original analytic dataset.

SUPPLEMENTARY FILE S6 Machine-readable question set and complete chatbot responses.

SUPPLEMENTARY FILE S7 R analysis code.
