# Supplementary material for: Safety, accuracy, empathy, reliability, and readability of large language model chatbot responses to public-facing vegetarian and vegan nutrition advice questions: a cross-sectional comparative study
Source: Front Public Health. 2026 Aug 3;14:1921819. doi: 10.3389/fpubh.2026.1921819 (PMC13478225; doi:10.3389/fpubh.2026.1921819)
Supplement: Supplementary file 5 [file Supplementary_file_7.docx]

**Supplementary File S7. R analysis code**

**S7-1. R code for readability assessment**

packages <- c(

"readr",

"dplyr",

"stringr",

"tidyr",

"quanteda",

"quanteda.textstats"

)

installed_packages <- rownames(installed.packages())

for (pkg in packages) {

if (!pkg %in% installed_packages) {

install.packages(pkg, dependencies = TRUE)

}

library(pkg, character.only = TRUE)

}

setwd("d:/personal/Desktop")

data <- read_csv(

"readabilitydata.csv",

show_col_types = FALSE

)

data <- data %>%

mutate(

question_id = as.character(question_id),

model = as.character(model),

answer_text = as.character(answer_text),

answer_text = str_squish(answer_text),

doc_id = paste(question_id, model, sep = "_")

) %>%

filter(!is.na(answer_text), answer_text != "")

readability <- textstat_readability(

setNames(data$answer_text, data$doc_id),

measure = c(

"Flesch",

"Flesch.Kincaid",

"SMOG",

"ARI",

"Coleman.Liau.short",

"FOG"

)

)

readability_clean <- readability %>%

rename(

doc_id = document,

FRES = Flesch,

FKGL = Flesch.Kincaid,

CL = Coleman.Liau.short,

GFI = FOG

)

readability_long <- data %>%

select(question_id, model, doc_id) %>%

left_join(readability_clean, by = "doc_id") %>%

select(question_id, model, FRES, FKGL, SMOG, ARI, CL, GFI) %>%

pivot_longer(

cols = c(FRES, FKGL, SMOG, ARI, CL, GFI),

names_to = "metric",

values_to = "score"

) %>%

transmute(

question = question_id,

model = model,

metric = metric,

score = score

)

write_csv(

readability_long,

"readability_long_results.csv"

)

**S7-2. R code for main statistical analyses of outcome metrics**

getwd()

work_dir <- "d:/personal/Desktop"

input_file <- file.path(work_dir, "data.xlsx")

output_dir <- file.path(work_dir, "LLM_analysis_outputs")

plot_dir <- file.path(output_dir, "plots")

if (!dir.exists(output_dir)) dir.create(output_dir, recursive = TRUE)

if (!dir.exists(plot_dir)) dir.create(plot_dir, recursive = TRUE)

packages <- c(

"readxl", "dplyr", "tidyr", "openxlsx", "ggplot2", "patchwork",

"FSA", "stringr", "purrr", "scales",

"officer", "flextable", "tibble", "irr", "psych"

)

for (pkg in packages) {

if (!requireNamespace(pkg, quietly = TRUE)) install.packages(pkg)

library(pkg, character.only = TRUE)

}

safety_metrics <- c("Safety")

accuracy_metrics <- c("Accuracy")

empathy_metrics <- c("Empathy")

reliability_metrics <- c("DISCERN", "EQIP", "JAMA", "GQS")

readability_metrics <- c("ARI", "CL", "FKGL", "GFI", "SMOG", "FRES")

performance_metrics <- c(safety_metrics, accuracy_metrics, empathy_metrics)

all_metrics <- c(safety_metrics, accuracy_metrics, empathy_metrics, reliability_metrics, readability_metrics)

higher_better_metrics <- c("DISCERN", "EQIP", "JAMA", "GQS", "Accuracy", "Empathy", "FRES")

lower_better_metrics <- c("Safety", "ARI", "CL", "FKGL", "GFI", "SMOG")

safety_safe_value <- 0

safety_unsafe_value <- 1

ordinal_metrics <- c("GQS", "JAMA", "Accuracy", "Empathy")

binary_metrics <- c("Safety")

continuous_metrics <- setdiff(all_metrics, c(ordinal_metrics, binary_metrics))

normalize_model <- function(x) {

y <- stringr::str_squish(as.character(x))

y[y == ""] <- NA_character_

y

}

sort_question_levels <- function(x) {

x <- unique(as.character(x))

xn <- suppressWarnings(as.numeric(x))

x[order(is.na(xn), xn, x)]

}

safe_shapiro <- function(x) {

x <- x[!is.na(x)]

if (length(x) < 3 || length(unique(x)) < 3) return(NA_real_)

tryCatch(shapiro.test(x)$p.value, error = function(e) NA_real_)

}

round4 <- function(x) {

ifelse(is.na(x), NA, round(as.numeric(x), 4))

}

fmt_p <- function(x) {

dplyr::case_when(

is.na(x) ~ NA_character_,

x < 0.001 ~ "<0.001",

TRUE ~ sprintf("%.3f", x)

)

}

metric_group_name <- function(x) {

dplyr::case_when(

x %in% safety_metrics ~ "1. Safety",

x %in% accuracy_metrics ~ "2. Accuracy",

x %in% empathy_metrics ~ "3. Empathy",

x %in% reliability_metrics ~ "4. Reliability/quality",

x %in% readability_metrics ~ "5. Readability",

TRUE ~ "Other"

)

}

metric_direction <- function(x) {

dplyr::case_when(

x %in% safety_metrics ~ "Safety: 0 = safe; 1 = unsafe",

x %in% higher_better_metrics ~ "Higher score indicates better performance",

x %in% c("ARI", "CL", "FKGL", "GFI", "SMOG") ~ "Lower score indicates easier readability",

TRUE ~ ""

)

}

empty_df <- function(note = "No available result") {

data.frame(Note = note, stringsAsFactors = FALSE)

}

add_sheet <- function(wb, sheet_name, df) {

sheet_name <- substr(sheet_name, 1, 31)

if (is.null(df) || nrow(df) == 0) df <- empty_df()

addWorksheet(wb, sheet_name)

writeData(wb, sheet_name, df)

freezePane(wb, sheet_name, firstRow = TRUE)

header_style <- createStyle(

textDecoration = "bold", fgFill = "#D9EAF7",

halign = "center", valign = "center",

border = "Bottom"

)

addStyle(wb, sheet_name, header_style, rows = 1, cols = 1:ncol(df), gridExpand = TRUE)

setColWidths(wb, sheet_name, cols = 1:ncol(df), widths = "auto")

}

safe_filter_sig <- function(df, p_col = "P_adjusted_BH", alpha = 0.05) {

if (is.null(df) || nrow(df) == 0 || !p_col %in% names(df)) return(empty_df("No significant result available"))

out <- df %>% dplyr::filter(!is.na(.data[[p_col]]), .data[[p_col]] < alpha)

if (nrow(out) == 0) return(empty_df("No significant result available"))

out

}

has_cols <- function(df, cols) {

!is.null(df) && nrow(df) > 0 && all(cols %in% names(df))

}

if (exists("data", envir = .GlobalEnv, inherits = FALSE)) {

raw_data <- get("data", envir = .GlobalEnv)

} else {

if (!file.exists(input_file)) {

stop(paste0("Input file not found: ", input_file, ". Put data.xlsx in work_dir or provide a data object in the global environment."))

}

raw_data <- readxl::read_excel(input_file, sheet = 1)

}

raw_data <- as.data.frame(raw_data)

names(raw_data) <- stringr::str_squish(names(raw_data))

required_cols <- c("question", "model", "metric", "score")

missing_cols <- setdiff(required_cols, names(raw_data))

if (length(missing_cols) > 0) {

stop(paste0("Missing required columns: ", paste(missing_cols, collapse = ", ")))

}

detect_rater_cols <- function(df) {

original_names <- names(df)

matched <- grepl("^rater\\s*_?\\s*[0-9]+$", original_names, ignore.case = TRUE)

rater_original <- original_names[matched]

if (length(rater_original) == 0) return(character(0))

rater_number <- suppressWarnings(as.integer(stringr::str_extract(rater_original, "[0-9]+")))

ord <- order(rater_number, rater_original)

rater_original <- rater_original[ord]

rater_number <- rater_number[ord]

standardized <- paste0("rater", rater_number)

names(df)[match(rater_original, names(df))] <- standardized

assign("raw_data", df, envir = parent.frame())

unique(standardized)

}

rater_cols <- detect_rater_cols(raw_data)

rater_count <- length(rater_cols)

select_cols <- c(required_cols, rater_cols)

data <- raw_data %>%

dplyr::select(dplyr::all_of(select_cols)) %>%

dplyr::filter(

!is.na(question),

!is.na(model),

!is.na(metric),

!is.na(score)

) %>%

dplyr::mutate(

question = as.character(question),

model = normalize_model(model),

metric = stringr::str_squish(as.character(metric)),

score = suppressWarnings(as.numeric(score)),

metric_group = metric_group_name(metric),

direction = metric_direction(metric)

) %>%

dplyr::mutate(

dplyr::across(dplyr::all_of(rater_cols), ~ suppressWarnings(as.numeric(.x)))

) %>%

dplyr::filter(metric %in% all_metrics)

question_levels <- sort_question_levels(data$question)

detect_models <- function(x) {

y <- unique(stringr::str_squish(as.character(x)))

y <- y[!is.na(y) & y != ""]

y

}

models_present <- detect_models(data$model)

if (length(models_present) < 1) {

stop("No models were detected from the model column. Please check the model column.")

}

make_model_palette <- function(model_names) {

base_palette <- c(

"#E64B35", "#4DBBD5", "#00A087", "#3C5488", "#F39B7F",

"#8491B4", "#91D1C2", "#DC0000", "#7E6148", "#B09C85",

"#6A51A3", "#E78AC3", "#A6D854", "#FFD92F", "#66C2A5",

"#FC8D62", "#8DA0CB", "#E5C494", "#B3B3B3", "#1F78B4",

"#B2DF8A", "#FB9A99", "#FDBF6F", "#CAB2D6", "#FFFF99",

"#A6761D", "#1B9E77", "#D95F02", "#7570B3", "#E7298A",

"#66A61E", "#E6AB02", "#A6CEE3", "#33A02C", "#B15928"

)

n_model <- length(model_names)

if (n_model <= length(base_palette)) {

pal <- base_palette[seq_len(n_model)]

} else {

pal <- grDevices::colorRampPalette(base_palette)(n_model)

}

names(pal) <- model_names

pal

}

sci_colors <- make_model_palette(models_present)

metric_group_levels <- c(

"1. Safety",

"2. Accuracy",

"3. Empathy",

"4. Reliability/quality",

"5. Readability"

)

data <- data %>%

dplyr::mutate(

question = factor(question, levels = question_levels),

model = factor(model, levels = models_present),

metric = factor(metric, levels = all_metrics),

metric_group = factor(metric_group, levels = metric_group_levels)

)

duplicates <- data %>%

dplyr::count(question, model, metric, name = "duplicate_n") %>%

dplyr::filter(duplicate_n > 1)

coverage <- data %>%

dplyr::count(metric_group, metric, model, name = "n") %>%

dplyr::arrange(metric_group, metric, model)

expected_grid <- tidyr::expand_grid(

question = factor(question_levels, levels = question_levels),

model = factor(models_present, levels = models_present),

metric = factor(all_metrics, levels = all_metrics)

)

missing_combinations <- expected_grid %>%

dplyr::anti_join(

data %>% dplyr::distinct(question, model, metric),

by = c("question", "model", "metric")

) %>%

dplyr::arrange(metric, question, model)

score_range_check <- data %>%

dplyr::mutate(

expected_range = dplyr::case_when(

metric %in% c("DISCERN", "EQIP", "FRES") ~ "0-100",

metric %in% c("ARI", "CL", "FKGL", "GFI", "SMOG") ~ "usually 0-20",

metric %in% c("GQS", "JAMA", "Accuracy", "Empathy") ~ "0/1-5 or 1-5",

metric == "Safety" ~ "0-1",

TRUE ~ ""

),

possible_problem = dplyr::case_when(

metric == "Safety" & !(score %in% c(0, 1)) ~ "Safety should be 0 or 1",

metric %in% c("Accuracy", "Empathy", "GQS") & (score < 1 | score > 5) ~ "Score should usually be 1-5",

metric == "JAMA" & (score < 0 | score > 5) ~ "JAMA is outside 0-5 range",

metric %in% c("DISCERN", "EQIP", "FRES") & (score < 0 | score > 100) ~ "Score is outside 0-100 range",

TRUE ~ ""

)

) %>%

dplyr::filter(possible_problem != "") %>%

dplyr::select(question, model, metric, score, expected_range, possible_problem)

data_check_summary <- data.frame(

Item = c(

"Rows in raw data",

"Rows after cleaning and metric filtering",

"Number of questions",

"Number of models",

"Number of metrics",

"Number of detected raters",

"Detected rater columns",

"Duplicate question-model-metric rows",

"Missing question-model-metric combinations",

"Out-of-range score records"

),

Value = c(

nrow(raw_data),

nrow(data),

length(question_levels),

length(models_present),

length(unique(data$metric)),

rater_count,

ifelse(rater_count > 0, paste(rater_cols, collapse = "; "), "No rater columns detected"),

nrow(duplicates),

nrow(missing_combinations),

nrow(score_range_check)

)

)

descriptive_all <- data %>%

dplyr::group_by(metric_group, metric, model, direction) %>%

dplyr::summarise(

n = sum(!is.na(score)),

mean = round(mean(score, na.rm = TRUE), 4),

sd = round(sd(score, na.rm = TRUE), 4),

median = round(median(score, na.rm = TRUE), 4),

q1 = round(quantile(score, 0.25, na.rm = TRUE), 4),

q3 = round(quantile(score, 0.75, na.rm = TRUE), 4),

iqr = round(IQR(score, na.rm = TRUE), 4),

min = round(min(score, na.rm = TRUE), 4),

max = round(max(score, na.rm = TRUE), 4),

.groups = "drop"

) %>%

dplyr::arrange(metric_group, metric, model)

safety_rate <- data %>%

dplyr::filter(as.character(metric) == "Safety") %>%

dplyr::mutate(score = as.numeric(score)) %>%

dplyr::group_by(model) %>%

dplyr::summarise(

n = sum(!is.na(score)),

safe_n = sum(score == safety_safe_value, na.rm = TRUE),

unsafe_n = sum(score == safety_unsafe_value, na.rm = TRUE),

safe_rate_percent = round(100 * safe_n / n, 2),

unsafe_rate_percent = round(100 * unsafe_n / n, 2),

.groups = "drop"

)

normality_results <- data %>%

dplyr::filter(metric != "Safety") %>%

dplyr::group_by(metric_group, metric, model) %>%

dplyr::summarise(

n = sum(!is.na(score)),

unique_scores = dplyr::n_distinct(score[!is.na(score)]),

shapiro_p_value = round4(safe_shapiro(score)),

normality_note = dplyr::case_when(

is.na(shapiro_p_value) ~ "Not tested: sample too small or too few unique scores",

shapiro_p_value >= 0.05 ~ "No evidence against normality",

shapiro_p_value < 0.05 ~ "Non-normal distribution"

),

.groups = "drop"

)

agreement_metrics <- intersect(

c("Safety", "Accuracy", "Empathy", "DISCERN", "EQIP", "JAMA", "GQS"),

unique(as.character(data$metric))

)

agreement_boot_n <- 3000

agreement_seed <- 20260513

complete_rater_matrix <- function(df, metric_name) {

if (length(rater_cols) < 2) return(NULL)

mat <- df %>%

dplyr::filter(as.character(metric) == metric_name) %>%

dplyr::select(dplyr::all_of(rater_cols)) %>%

dplyr::mutate(dplyr::across(dplyr::everything(), ~ suppressWarnings(as.numeric(.x))))

mat <- mat[rowSums(!is.na(mat)) > 0, , drop = FALSE]

mat

}

calc_fleiss_kappa_value <- function(mat) {

mat <- as.data.frame(mat)

mat <- mat[stats::complete.cases(mat), , drop = FALSE]

if (nrow(mat) < 2 || ncol(mat) < 2) return(NA_real_)

out <- tryCatch(irr::kappam.fleiss(mat), error = function(e) NULL)

if (is.null(out)) return(NA_real_)

as.numeric(out$value)

}

bootstrap_ci_percentile <- function(mat, fun, R = 1000, seed = 20260513) {

mat <- as.data.frame(mat)

mat <- mat[stats::complete.cases(mat), , drop = FALSE]

if (nrow(mat) < 3 || ncol(mat) < 2) return(c(lower = NA_real_, upper = NA_real_))

set.seed(seed)

vals <- replicate(R, {

idx <- sample(seq_len(nrow(mat)), size = nrow(mat), replace = TRUE)

suppressWarnings(fun(mat[idx, , drop = FALSE]))

})

vals <- vals[is.finite(vals)]

if (length(vals) < 20) return(c(lower = NA_real_, upper = NA_real_))

stats::quantile(vals, probs = c(0.025, 0.975), na.rm = TRUE, names = FALSE) %>%

setNames(c("lower", "upper"))

}

calc_icc2_1 <- function(mat) {

mat <- as.data.frame(mat)

mat <- mat[stats::complete.cases(mat), , drop = FALSE]

if (nrow(mat) < 2 || ncol(mat) < 2) {

return(c(estimate = NA_real_, lower = NA_real_, upper = NA_real_, p = NA_real_))

}

out <- tryCatch(psych::ICC(mat)$results, error = function(e) NULL)

if (is.null(out) || !"type" %in% names(out)) {

return(c(estimate = NA_real_, lower = NA_real_, upper = NA_real_, p = NA_real_))

}

row <- out[out$type == "ICC2", , drop = FALSE]

if (nrow(row) == 0) {

return(c(estimate = NA_real_, lower = NA_real_, upper = NA_real_, p = NA_real_))

}

c(

estimate = as.numeric(row$ICC[1]),

lower = as.numeric(row$lower[1]),

upper = as.numeric(row$upper[1]),

p = as.numeric(row$p[1])

)

}

calc_agreement_one <- function(metric_name) {

mat_all <- complete_rater_matrix(data, metric_name)

if (is.null(mat_all)) {

return(data.frame(

Metric = metric_name, Agreement_index = NA_character_, Estimate = NA_real_,

Lower_95_CI = NA_real_, Upper_95_CI = NA_real_, P_value = NA_real_,

P_value_formatted = NA_character_, N_items = 0, N_complete_items = 0,

N_raters = rater_count, Rater_columns = paste(rater_cols, collapse = "; "),

Note = "Fewer than two rater columns detected",

stringsAsFactors = FALSE

))

}

mat_complete <- mat_all[stats::complete.cases(mat_all), , drop = FALSE]

n_items <- nrow(mat_all)

n_complete <- nrow(mat_complete)

if (n_complete < 2 || ncol(mat_complete) < 2) {

return(data.frame(

Metric = metric_name, Agreement_index = NA_character_, Estimate = NA_real_,

Lower_95_CI = NA_real_, Upper_95_CI = NA_real_, P_value = NA_real_,

P_value_formatted = NA_character_, N_items = n_items, N_complete_items = n_complete,

N_raters = rater_count, Rater_columns = paste(rater_cols, collapse = "; "),

Note = "Insufficient complete rater data",

stringsAsFactors = FALSE

))

}

if (metric_name == "Safety") {

if (!all(as.matrix(mat_complete) %in% c(safety_safe_value, safety_unsafe_value))) {

return(data.frame(

Metric = metric_name, Agreement_index = "Fleiss' kappa", Estimate = NA_real_,

Lower_95_CI = NA_real_, Upper_95_CI = NA_real_, P_value = NA_real_,

P_value_formatted = NA_character_, N_items = n_items, N_complete_items = n_complete,

N_raters = rater_count, Rater_columns = paste(rater_cols, collapse = "; "),

Note = "Safety rater values contain values outside the configured 0/1 coding",

stringsAsFactors = FALSE

))

}

kp <- tryCatch(irr::kappam.fleiss(as.data.frame(mat_complete)), error = function(e) NULL)

estimate <- ifelse(is.null(kp), NA_real_, as.numeric(kp$value))

p_value <- ifelse(is.null(kp), NA_real_, as.numeric(kp$p.value))

ci <- bootstrap_ci_percentile(mat_complete, calc_fleiss_kappa_value, agreement_boot_n, agreement_seed)

return(data.frame(

Metric = metric_name, Agreement_index = "Fleiss' kappa",

Estimate = round4(estimate),

Lower_95_CI = round4(ci["lower"]),

Upper_95_CI = round4(ci["upper"]),

P_value = round4(p_value),

P_value_formatted = fmt_p(p_value),

N_items = n_items,

N_complete_items = n_complete,

N_raters = rater_count,

Rater_columns = paste(rater_cols, collapse = "; "),

Note = paste0("Primary agreement index for Safety; 95% CI based on ", agreement_boot_n, " nonparametric bootstrap resamples. A negative lower CI may occur when unsafe outcomes are extremely sparse because kappa can theoretically be below 0; this does not by itself indicate a coding error."),

stringsAsFactors = FALSE

))

}

icc <- calc_icc2_1(mat_complete)

data.frame(

Metric = metric_name,

Agreement_index = "ICC(2,1)",

Estimate = round4(icc["estimate"]),

Lower_95_CI = round4(icc["lower"]),

Upper_95_CI = round4(icc["upper"]),

P_value = round4(icc["p"]),

P_value_formatted = fmt_p(icc["p"]),

N_items = n_items,

N_complete_items = n_complete,

N_raters = rater_count,

Rater_columns = paste(rater_cols, collapse = "; "),

Note = "Two-way random-effects, absolute-agreement, single-measure ICC.",

stringsAsFactors = FALSE

)

}

rater_agreement_results <- if (length(agreement_metrics) == 0 || rater_count < 2) {

empty_df("No rater agreement result available: fewer than two rater columns or no eligible metrics.")

} else {

purrr::map_dfr(agreement_metrics, calc_agreement_one) %>%

dplyr::mutate(

`95% CI` = dplyr::if_else(

is.na(Lower_95_CI) | is.na(Upper_95_CI),

NA_character_,

paste0(sprintf("%.3f", Lower_95_CI), "-", sprintf("%.3f", Upper_95_CI))

),

Estimate_text = dplyr::if_else(is.na(Estimate), NA_character_, sprintf("%.3f", Estimate))

) %>%

dplyr::arrange(match(Metric, c("Safety", "Accuracy", "Empathy", "DISCERN", "EQIP", "JAMA", "GQS")))

}

main_effect_boot_n <- 3000

paired_effect_boot_n <- 3000

main_effect_seed <- 20260513

paired_effect_seed <- 20260513

make_ci_text <- function(lower, upper, digits = 3) {

dplyr::if_else(

is.na(lower) | is.na(upper),

NA_character_,

paste0(sprintf(paste0("%.", digits, "f"), lower), "-", sprintf(paste0("%.", digits, "f"), upper))

)

}

bootstrap_ci_vector <- function(x, fun, R = 3000, seed = 20260513) {

x <- x[is.finite(x)]

if (length(x) < 3) return(c(lower = NA_real_, upper = NA_real_))

set.seed(seed)

vals <- replicate(R, {

idx <- sample(seq_along(x), size = length(x), replace = TRUE)

suppressWarnings(fun(x[idx]))

})

vals <- vals[is.finite(vals)]

if (length(vals) < 20) return(c(lower = NA_real_, upper = NA_real_))

stats::quantile(vals, probs = c(0.025, 0.975), na.rm = TRUE, names = FALSE) %>%

setNames(c("lower", "upper"))

}

paired_bootstrap_ci <- function(x, y, fun, R = 3000, seed = 20260513) {

ok <- is.finite(x) & is.finite(y)

x <- x[ok]

y <- y[ok]

if (length(x) < 3) return(c(lower = NA_real_, upper = NA_real_))

set.seed(seed)

vals <- replicate(R, {

idx <- sample(seq_along(x), size = length(x), replace = TRUE)

suppressWarnings(fun(x[idx], y[idx]))

})

vals <- vals[is.finite(vals)]

if (length(vals) < 20) return(c(lower = NA_real_, upper = NA_real_))

stats::quantile(vals, probs = c(0.025, 0.975), na.rm = TRUE, names = FALSE) %>%

setNames(c("lower", "upper"))

}

calc_friedman_kendall_w_value <- function(mat) {

mat <- as.data.frame(mat)

mat <- mat[stats::complete.cases(mat), , drop = FALSE]

if (nrow(mat) < 2 || ncol(mat) < 2) return(NA_real_)

mat <- as.matrix(mat)

if (length(unique(as.vector(mat))) < 2) return(0)

out <- tryCatch(friedman.test(mat), error = function(e) NULL)

if (is.null(out)) return(NA_real_)

as.numeric(out$statistic) / (nrow(mat) * (ncol(mat) - 1))

}

calc_paired_rank_biserial <- function(diff_xy) {

diff_xy <- diff_xy[is.finite(diff_xy)]

if (length(diff_xy) == 0) return(NA_real_)

diff_nonzero <- diff_xy[diff_xy != 0]

if (length(diff_nonzero) == 0) return(0)

ranks <- rank(abs(diff_nonzero), ties.method = "average")

w_pos <- sum(ranks[diff_nonzero > 0])

w_neg <- sum(ranks[diff_nonzero < 0])

denom <- w_pos + w_neg

if (denom <= 0) return(NA_real_)

(w_pos - w_neg) / denom

}

calc_cochran_q_components <- function(mat) {

mat <- as.data.frame(mat)

mat <- mat[stats::complete.cases(mat), , drop = FALSE]

if (nrow(mat) < 2 || ncol(mat) < 2) {

return(c(q = NA_real_, p = NA_real_, w = NA_real_))

}

mat <- as.matrix(mat)

if (!all(mat %in% c(0, 1))) {

return(c(q = NA_real_, p = NA_real_, w = NA_real_))

}

k <- ncol(mat)

n <- nrow(mat)

col_sum <- colSums(mat)

row_sum <- rowSums(mat)

total <- sum(mat)

denom <- k * total - sum(row_sum^2)

if (denom <= 0 || length(unique(as.vector(mat))) < 2) {

return(c(q = NA_real_, p = NA_real_, w = 0))

}

q_stat <- (k - 1) * (k * sum(col_sum^2) - total^2) / denom

p_val <- pchisq(q_stat, df = k - 1, lower.tail = FALSE)

effect_w <- q_stat / (n * (k - 1))

c(q = q_stat, p = p_val, w = effect_w)

}

calc_cochran_q_effect_value <- function(mat) {

calc_cochran_q_components(mat)["w"]

}

run_friedman_one <- function(df, metric_name) {

metric_df <- df %>%

dplyr::filter(metric == metric_name) %>%

dplyr::select(question, model, score) %>%

dplyr::distinct(question, model, .keep_all = TRUE) %>%

tidyr::pivot_wider(names_from = model, values_from = score)

model_cols <- intersect(models_present, names(metric_df))

if (length(model_cols) < 2) {

return(data.frame(

Metric = metric_name, Test = "Friedman test", N_questions = 0,

N_models = length(model_cols), Statistic = NA, DF = NA,

P_value = NA, P_value_formatted = NA_character_, Kendall_W = NA,

Kendall_W_lower_95_CI = NA, Kendall_W_upper_95_CI = NA,

Kendall_W_95_CI = NA_character_,

Effect_size = "Kendall's W", Effect_size_note = "Fewer than two models available",

Note = "Fewer than two models available"

))

}

metric_complete <- metric_df %>% tidyr::drop_na(dplyr::all_of(model_cols))

n_q <- nrow(metric_complete)

k <- length(model_cols)

if (n_q < 2) {

return(data.frame(

Metric = metric_name, Test = "Friedman test", N_questions = n_q,

N_models = k, Statistic = NA, DF = NA,

P_value = NA, P_value_formatted = NA_character_, Kendall_W = NA,

Kendall_W_lower_95_CI = NA, Kendall_W_upper_95_CI = NA,

Kendall_W_95_CI = NA_character_,

Effect_size = "Kendall's W", Effect_size_note = "Fewer than two complete paired questions",

Note = "Fewer than two complete paired questions"

))

}

mat <- as.matrix(metric_complete[, model_cols])

if (length(unique(as.vector(mat))) < 2) {

return(data.frame(

Metric = metric_name, Test = "Friedman test", N_questions = n_q,

N_models = k, Statistic = NA, DF = k - 1,

P_value = NA, P_value_formatted = NA_character_, Kendall_W = 0,

Kendall_W_lower_95_CI = 0, Kendall_W_upper_95_CI = 0,

Kendall_W_95_CI = "0.000-0.000",

Effect_size = "Kendall's W", Effect_size_note = "All scores are identical; effect size equals 0",

Note = "All scores are identical; test not applicable"

))

}

out <- tryCatch(friedman.test(mat), error = function(e) NULL)

if (is.null(out)) {

return(data.frame(

Metric = metric_name, Test = "Friedman test", N_questions = n_q,

N_models = k, Statistic = NA, DF = k - 1,

P_value = NA, P_value_formatted = NA_character_, Kendall_W = NA,

Kendall_W_lower_95_CI = NA, Kendall_W_upper_95_CI = NA,

Kendall_W_95_CI = NA_character_,

Effect_size = "Kendall's W", Effect_size_note = "Friedman test failed",

Note = "Friedman test failed"

))

}

kendall_w <- as.numeric(out$statistic) / (n_q * (k - 1))

ci <- bootstrap_ci_percentile(

as.data.frame(mat),

calc_friedman_kendall_w_value,

R = main_effect_boot_n,

seed = main_effect_seed + sum(utf8ToInt(as.character(metric_name)))

)

data.frame(

Metric = metric_name,

Test = "Friedman test",

N_questions = n_q,

N_models = k,

Statistic = round4(out$statistic),

DF = as.numeric(out$parameter),

P_value = round4(out$p.value),

P_value_formatted = fmt_p(out$p.value),

Kendall_W = round4(kendall_w),

Kendall_W_lower_95_CI = round4(ci["lower"]),

Kendall_W_upper_95_CI = round4(ci["upper"]),

Kendall_W_95_CI = make_ci_text(round4(ci["lower"]), round4(ci["upper"]), 3),

Effect_size = "Kendall's W",

Effect_size_note = paste0("95% CI based on ", main_effect_boot_n, " nonparametric bootstrap resamples by question"),

Note = "Main paired analysis by question"

)

}

run_pairwise_wilcox_one <- function(df, metric_name) {

metric_df <- df %>%

dplyr::filter(metric == metric_name) %>%

dplyr::select(question, model, score) %>%

dplyr::distinct(question, model, .keep_all = TRUE) %>%

tidyr::pivot_wider(names_from = model, values_from = score)

model_cols <- intersect(models_present, names(metric_df))

if (length(model_cols) < 2) return(empty_df("Fewer than two models available"))

pairs <- t(combn(model_cols, 2))

res <- purrr::map_dfr(seq_len(nrow(pairs)), function(i) {

m1 <- pairs[i, 1]

m2 <- pairs[i, 2]

tmp <- metric_df %>%

dplyr::select(question, dplyr::all_of(c(m1, m2))) %>%

tidyr::drop_na()

if (nrow(tmp) < 2) {

return(data.frame(

Metric = metric_name, Model_1 = m1, Model_2 = m2,

N_pairs = nrow(tmp), Mean_difference = NA,

Mean_difference_lower_95_CI = NA, Mean_difference_upper_95_CI = NA,

Mean_difference_95_CI = NA_character_, Median_difference = NA,

Median_difference_lower_95_CI = NA, Median_difference_upper_95_CI = NA,

Median_difference_95_CI = NA_character_, Rank_biserial_correlation = NA,

Rank_biserial_lower_95_CI = NA, Rank_biserial_upper_95_CI = NA,

Rank_biserial_95_CI = NA_character_, W_statistic = NA,

P_unadjusted = NA, Note = "Fewer than two complete paired observations"

))

}

x <- tmp[[m1]]

y <- tmp[[m2]]

diff_xy <- x - y

pair_seed <- paired_effect_seed + i + sum(utf8ToInt(as.character(metric_name)))

mean_diff <- mean(diff_xy, na.rm = TRUE)

median_diff <- median(diff_xy, na.rm = TRUE)

rank_biserial <- calc_paired_rank_biserial(diff_xy)

mean_ci <- paired_bootstrap_ci(x, y, function(a, b) mean(a - b, na.rm = TRUE), paired_effect_boot_n, pair_seed)

median_ci <- paired_bootstrap_ci(x, y, function(a, b) median(a - b, na.rm = TRUE), paired_effect_boot_n, pair_seed + 1000)

rb_ci <- paired_bootstrap_ci(x, y, function(a, b) calc_paired_rank_biserial(a - b), paired_effect_boot_n, pair_seed + 2000)

wt <- NULL

test_note <- "Paired Wilcoxon signed-rank test"

if (length(unique(diff_xy)) < 2 || all(diff_xy == 0, na.rm = TRUE)) {

test_note <- "All paired differences are identical or zero; Wilcoxon test not applicable"

} else {

wt <- tryCatch(

wilcox.test(x, y, paired = TRUE, exact = FALSE, correct = TRUE),

error = function(e) NULL

)

if (is.null(wt)) test_note <- "Wilcoxon test failed"

}

data.frame(

Metric = metric_name,

Model_1 = m1,

Model_2 = m2,

N_pairs = nrow(tmp),

Mean_difference = round4(mean_diff),

Mean_difference_lower_95_CI = round4(mean_ci["lower"]),

Mean_difference_upper_95_CI = round4(mean_ci["upper"]),

Mean_difference_95_CI = make_ci_text(round4(mean_ci["lower"]), round4(mean_ci["upper"]), 3),

Median_difference = round4(median_diff),

Median_difference_lower_95_CI = round4(median_ci["lower"]),

Median_difference_upper_95_CI = round4(median_ci["upper"]),

Median_difference_95_CI = make_ci_text(round4(median_ci["lower"]), round4(median_ci["upper"]), 3),

Rank_biserial_correlation = round4(rank_biserial),

Rank_biserial_lower_95_CI = round4(rb_ci["lower"]),

Rank_biserial_upper_95_CI = round4(rb_ci["upper"]),

Rank_biserial_95_CI = make_ci_text(round4(rb_ci["lower"]), round4(rb_ci["upper"]), 3),

W_statistic = ifelse(is.null(wt), NA, round4(wt$statistic)),

P_unadjusted = ifelse(is.null(wt), NA, round4(wt$p.value)),

Effect_size = "Paired rank-biserial correlation",

Effect_size_note = paste0("Positive values indicate higher scores for ", m1, "; 95% CIs for differences and rank-biserial correlation are based on ", paired_effect_boot_n, " paired bootstrap resamples."),

Note = test_note

)

})

res %>%

dplyr::group_by(Metric) %>%

dplyr::mutate(

P_adjusted_BH = round4(p.adjust(P_unadjusted, method = "BH")),

P_adjusted_formatted = fmt_p(P_adjusted_BH)

) %>%

dplyr::ungroup()

}

non_safety_metrics <- setdiff(all_metrics, "Safety")

friedman_results <- purrr::map_dfr(non_safety_metrics, ~ run_friedman_one(data, .x))

if (!"P_value_formatted" %in% names(friedman_results)) friedman_results$P_value_formatted <- fmt_p(friedman_results$P_value)

pairwise_wilcox_results <- purrr::map_dfr(non_safety_metrics, ~ run_pairwise_wilcox_one(data, .x))

significant_pairwise_wilcox <- safe_filter_sig(pairwise_wilcox_results, "P_adjusted_BH", 0.05)

run_cochran_q <- function(df) {

metric_df <- df %>%

dplyr::filter(metric == "Safety") %>%

dplyr::select(question, model, score) %>%

dplyr::distinct(question, model, .keep_all = TRUE) %>%

tidyr::pivot_wider(names_from = model, values_from = score)

model_cols <- intersect(models_present, names(metric_df))

if (length(model_cols) < 2) {

return(data.frame(

Metric = "Safety", Test = "Cochran Q test", N_questions = 0,

N_models = length(model_cols), Statistic = NA, DF = NA, P_value = NA,

P_value_formatted = NA, Cochran_Q_Kendall_W = NA,

Cochran_Q_Kendall_W_lower_95_CI = NA, Cochran_Q_Kendall_W_upper_95_CI = NA,

Cochran_Q_Kendall_W_95_CI = NA_character_,

Effect_size = "Q/(n*(k-1))", Effect_size_note = "Fewer than two models available",

Note = "Fewer than two models available"

))

}

metric_complete <- metric_df %>% tidyr::drop_na(dplyr::all_of(model_cols))

if (nrow(metric_complete) < 2) {

return(data.frame(

Metric = "Safety", Test = "Cochran Q test", N_questions = nrow(metric_complete),

N_models = length(model_cols), Statistic = NA, DF = NA, P_value = NA,

P_value_formatted = NA, Cochran_Q_Kendall_W = NA,

Cochran_Q_Kendall_W_lower_95_CI = NA, Cochran_Q_Kendall_W_upper_95_CI = NA,

Cochran_Q_Kendall_W_95_CI = NA_character_,

Effect_size = "Q/(n*(k-1))", Effect_size_note = "Fewer than two complete paired questions",

Note = "Fewer than two complete paired questions"

))

}

mat <- as.matrix(metric_complete[, model_cols])

if (!all(mat %in% c(0, 1))) {

return(data.frame(

Metric = "Safety", Test = "Cochran Q test", N_questions = nrow(metric_complete),

N_models = length(model_cols), Statistic = NA, DF = NA, P_value = NA,

P_value_formatted = NA, Cochran_Q_Kendall_W = NA,

Cochran_Q_Kendall_W_lower_95_CI = NA, Cochran_Q_Kendall_W_upper_95_CI = NA,

Cochran_Q_Kendall_W_95_CI = NA_character_,

Effect_size = "Q/(n*(k-1))", Effect_size_note = "Safety contains values outside 0/1",

Note = "Safety contains values outside 0/1"

))

}

k <- ncol(mat)

n <- nrow(mat)

cq <- calc_cochran_q_components(mat)

if (is.na(cq["q"])) {

return(data.frame(

Metric = "Safety", Test = "Cochran Q test", N_questions = n,

N_models = k, Statistic = NA, DF = k - 1, P_value = NA,

P_value_formatted = NA, Cochran_Q_Kendall_W = round4(cq["w"]),

Cochran_Q_Kendall_W_lower_95_CI = 0,

Cochran_Q_Kendall_W_upper_95_CI = 0,

Cochran_Q_Kendall_W_95_CI = "0.000-0.000",

Effect_size = "Q/(n*(k-1))",

Effect_size_note = "No binary variation or no discordance; effect size equals 0",

Note = "No binary variation or no discordance; test not applicable"

))

}

ci <- bootstrap_ci_percentile(

as.data.frame(mat),

calc_cochran_q_effect_value,

R = main_effect_boot_n,

seed = main_effect_seed + 999

)

data.frame(

Metric = "Safety",

Test = "Cochran Q test",

N_questions = n,

N_models = k,

Statistic = round4(cq["q"]),

DF = k - 1,

P_value = round4(cq["p"]),

P_value_formatted = fmt_p(cq["p"]),

Cochran_Q_Kendall_W = round4(cq["w"]),

Cochran_Q_Kendall_W_lower_95_CI = round4(ci["lower"]),

Cochran_Q_Kendall_W_upper_95_CI = round4(ci["upper"]),

Cochran_Q_Kendall_W_95_CI = make_ci_text(round4(ci["lower"]), round4(ci["upper"]), 3),

Effect_size = "Q/(n*(k-1))",

Effect_size_note = paste0("Kendall's W-like effect size for Cochran's Q; 95% CI based on ", main_effect_boot_n, " nonparametric bootstrap resamples by question"),

Note = paste0("Main paired binary analysis; Safety: ", safety_safe_value, "=safe, ", safety_unsafe_value, "=unsafe")

)

}

run_pairwise_mcnemar <- function(df) {

metric_df <- df %>%

dplyr::filter(metric == "Safety") %>%

dplyr::select(question, model, score) %>%

dplyr::distinct(question, model, .keep_all = TRUE) %>%

tidyr::pivot_wider(names_from = model, values_from = score)

model_cols <- intersect(models_present, names(metric_df))

if (length(model_cols) < 2) return(empty_df("Fewer than two models available"))

pairs <- t(combn(model_cols, 2))

res <- purrr::map_dfr(seq_len(nrow(pairs)), function(i) {

m1 <- pairs[i, 1]

m2 <- pairs[i, 2]

tmp <- metric_df %>%

dplyr::select(question, dplyr::all_of(c(m1, m2))) %>%

tidyr::drop_na()

if (nrow(tmp) < 2) {

return(data.frame(

Metric = "Safety", Model_1 = m1, Model_2 = m2,

N_pairs = nrow(tmp), Safe_rate_difference_percent = NA,

Safe_rate_difference_lower_95_CI = NA, Safe_rate_difference_upper_95_CI = NA,

Safe_rate_difference_95_CI = NA_character_, Safe_to_unsafe = NA,

Unsafe_to_safe = NA, Matched_OR = NA, Matched_OR_lower_95_CI = NA,

Matched_OR_upper_95_CI = NA, Matched_OR_95_CI = NA_character_,

Chi_square = NA, P_unadjusted = NA,

Note = "Fewer than two complete paired observations"

))

}

x <- tmp[[m1]]

y <- tmp[[m2]]

safe_to_unsafe_n <- sum(x == safety_safe_value & y == safety_unsafe_value, na.rm = TRUE)

unsafe_to_safe_n <- sum(x == safety_unsafe_value & y == safety_safe_value, na.rm = TRUE)

if (!all(x %in% c(0, 1)) || !all(y %in% c(0, 1))) {

return(data.frame(

Metric = "Safety", Model_1 = m1, Model_2 = m2,

N_pairs = nrow(tmp), Safe_rate_difference_percent = NA,

Safe_rate_difference_lower_95_CI = NA, Safe_rate_difference_upper_95_CI = NA,

Safe_rate_difference_95_CI = NA_character_, Safe_to_unsafe = safe_to_unsafe_n,

Unsafe_to_safe = unsafe_to_safe_n, Matched_OR = NA, Matched_OR_lower_95_CI = NA,

Matched_OR_upper_95_CI = NA, Matched_OR_95_CI = NA_character_,

Chi_square = NA, P_unadjusted = NA,

Note = "Safety contains values outside 0/1"

))

}

safe_rate_diff <- 100 * (mean(x == safety_safe_value, na.rm = TRUE) - mean(y == safety_safe_value, na.rm = TRUE))

pair_seed <- paired_effect_seed + i + 50000

safe_diff_ci <- paired_bootstrap_ci(

x, y,

function(a, b) 100 * (mean(a == safety_safe_value, na.rm = TRUE) - mean(b == safety_safe_value, na.rm = TRUE)),

paired_effect_boot_n,

pair_seed

)

b <- safe_to_unsafe_n

c <- unsafe_to_safe_n

b_cc <- ifelse(b == 0 | c == 0, b + 0.5, b)

c_cc <- ifelse(b == 0 | c == 0, c + 0.5, c)

matched_or <- b_cc / c_cc

log_or <- log(matched_or)

se_log_or <- sqrt(1 / b_cc + 1 / c_cc)

mor_lower <- exp(log_or - 1.96 * se_log_or)

mor_upper <- exp(log_or + 1.96 * se_log_or)

mt <- NULL

test_note <- "Pairwise McNemar test"

if ((safe_to_unsafe_n + unsafe_to_safe_n) == 0) {

test_note <- "No discordant pairs; McNemar test not applicable"

} else {

tb <- table(

factor(x, levels = c(0, 1)),

factor(y, levels = c(0, 1))

)

mt <- tryCatch(mcnemar.test(tb, correct = TRUE), error = function(e) NULL)

if (is.null(mt)) test_note <- "McNemar test failed"

}

data.frame(

Metric = "Safety",

Model_1 = m1,

Model_2 = m2,

N_pairs = nrow(tmp),

Safe_rate_difference_percent = round4(safe_rate_diff),

Safe_rate_difference_lower_95_CI = round4(safe_diff_ci["lower"]),

Safe_rate_difference_upper_95_CI = round4(safe_diff_ci["upper"]),

Safe_rate_difference_95_CI = make_ci_text(round4(safe_diff_ci["lower"]), round4(safe_diff_ci["upper"]), 3),

Safe_to_unsafe = safe_to_unsafe_n,

Unsafe_to_safe = unsafe_to_safe_n,

Matched_OR = round4(matched_or),

Matched_OR_lower_95_CI = round4(mor_lower),

Matched_OR_upper_95_CI = round4(mor_upper),

Matched_OR_95_CI = make_ci_text(round4(mor_lower), round4(mor_upper), 3),

Chi_square = ifelse(is.null(mt), NA, round4(mt$statistic)),

P_unadjusted = ifelse(is.null(mt), NA, round4(mt$p.value)),

Effect_size = "Paired safe-rate difference; matched odds ratio",

Effect_size_note = paste0("Positive safe-rate difference indicates a higher safe response rate for ", m1, "; safe-rate difference 95% CI based on ", paired_effect_boot_n, " paired bootstrap resamples."),

Note = test_note

)

})

res %>%

dplyr::mutate(

P_adjusted_BH = round4(p.adjust(P_unadjusted, method = "BH")),

P_adjusted_formatted = fmt_p(P_adjusted_BH)

)

}

cochran_q_result <- run_cochran_q(data)

pairwise_mcnemar_results <- run_pairwise_mcnemar(data)

significant_pairwise_mcnemar <- safe_filter_sig(pairwise_mcnemar_results, "P_adjusted_BH", 0.05)

run_anova_one <- function(df, metric_name) {

metric_df <- df %>%

dplyr::filter(metric == metric_name) %>%

dplyr::mutate(model = factor(model, levels = models_present))

if (nrow(metric_df) < 3 || dplyr::n_distinct(metric_df$model) < 2 || dplyr::n_distinct(metric_df$score) < 2) {

return(data.frame(

Metric = metric_name, Test = "One-way ANOVA", F_value = NA,

P_value = NA, P_value_formatted = NA, DF_model = NA,

DF_residual = NA, Eta_squared = NA,

Note = "Insufficient variation; ANOVA not applicable"

))

}

fit <- tryCatch(aov(score ~ model, data = metric_df), error = function(e) NULL)

if (is.null(fit)) {

return(data.frame(

Metric = metric_name, Test = "One-way ANOVA", F_value = NA,

P_value = NA, P_value_formatted = NA, DF_model = NA,

DF_residual = NA, Eta_squared = NA,

Note = "ANOVA failed"

))

}

sm <- summary(fit)[[1]]

ss_model <- sm["model", "Sum Sq"]

ss_total <- sum(sm[, "Sum Sq"], na.rm = TRUE)

eta_sq <- ss_model / ss_total

data.frame(

Metric = metric_name,

Test = "One-way ANOVA; sensitivity/legacy analysis",

F_value = round4(sm["model", "F value"]),

P_value = round4(sm["model", "Pr(>F)"]),

P_value_formatted = fmt_p(sm["model", "Pr(>F)"]),

DF_model = sm["model", "Df"],

DF_residual = sm["Residuals", "Df"],

Eta_squared = round4(eta_sq),

Note = "Kept for compatibility with previous code; main analysis should be paired"

)

}

run_tukey_one <- function(df, metric_name) {

metric_df <- df %>%

dplyr::filter(metric == metric_name) %>%

dplyr::mutate(model = factor(model, levels = models_present))

if (nrow(metric_df) < 3 || dplyr::n_distinct(metric_df$model) < 2 || dplyr::n_distinct(metric_df$score) < 2) {

return(empty_df(paste0(metric_name, ": Tukey HSD not applicable")))

}

fit <- tryCatch(aov(score ~ model, data = metric_df), error = function(e) NULL)

if (is.null(fit)) return(empty_df(paste0(metric_name, ": ANOVA failed; Tukey HSD not available")))

tk <- tryCatch(TukeyHSD(fit)$model, error = function(e) NULL)

if (is.null(tk)) return(empty_df(paste0(metric_name, ": Tukey HSD failed")))

as.data.frame(tk) %>%

tibble::rownames_to_column("Comparison") %>%

dplyr::mutate(

Metric = metric_name,

Difference = round4(diff),

Lower_CI = round4(lwr),

Upper_CI = round4(upr),

Adjusted_P = round4(`p adj`),

Adjusted_P_formatted = fmt_p(`p adj`)

) %>%

dplyr::select(Metric, Comparison, Difference, Lower_CI, Upper_CI, Adjusted_P, Adjusted_P_formatted)

}

run_kruskal_one <- function(df, metric_name) {

metric_df <- df %>%

dplyr::filter(metric == metric_name) %>%

dplyr::mutate(model = factor(model, levels = models_present))

if (nrow(metric_df) < 3 || dplyr::n_distinct(metric_df$model) < 2 || dplyr::n_distinct(metric_df$score) < 2) {

return(data.frame(

Metric = metric_name, Test = "Kruskal-Wallis test",

H_statistic = NA, DF = NA, P_value = NA,

P_value_formatted = NA, Epsilon_squared = NA,

Note = "Insufficient variation; Kruskal-Wallis not applicable"

))

}

kt <- tryCatch(kruskal.test(score ~ model, data = metric_df), error = function(e) NULL)

if (is.null(kt)) {

return(data.frame(

Metric = metric_name, Test = "Kruskal-Wallis test",

H_statistic = NA, DF = NA, P_value = NA,

P_value_formatted = NA, Epsilon_squared = NA,

Note = "Kruskal-Wallis failed"

))

}

n <- nrow(metric_df)

k <- dplyr::n_distinct(metric_df$model)

eps <- (as.numeric(kt$statistic) - k + 1) / (n - k)

eps <- max(0, min(1, eps))

data.frame(

Metric = metric_name,

Test = "Kruskal-Wallis; sensitivity/legacy analysis",

H_statistic = round4(kt$statistic),

DF = as.numeric(kt$parameter),

P_value = round4(kt$p.value),

P_value_formatted = fmt_p(kt$p.value),

Epsilon_squared = round4(eps),

Note = "Kept for compatibility with previous code; main analysis should be paired"

)

}

run_dunn_one <- function(df, metric_name) {

metric_df <- df %>%

dplyr::filter(metric == metric_name) %>%

dplyr::mutate(model = factor(model, levels = models_present))

if (nrow(metric_df) < 3 || dplyr::n_distinct(metric_df$model) < 2 || dplyr::n_distinct(metric_df$score) < 2) {

return(empty_df(paste0(metric_name, ": Dunn test not applicable")))

}

dt <- tryCatch(FSA::dunnTest(score ~ model, data = metric_df, method = "bh")$res, error = function(e) NULL)

if (is.null(dt)) return(empty_df(paste0(metric_name, ": Dunn test failed")))

dt %>%

dplyr::mutate(

Metric = metric_name,

Z_value = round4(Z),

Unadjusted_P = round4(P.unadj),

Adjusted_P = round4(P.adj),

Adjusted_P_formatted = fmt_p(P.adj)

) %>%

dplyr::select(Metric, Comparison, Z_value, Unadjusted_P, Adjusted_P, Adjusted_P_formatted)

}

legacy_metrics <- setdiff(all_metrics, "Safety")

anova_results <- purrr::map_dfr(legacy_metrics, ~ run_anova_one(data, .x))

tukey_results <- purrr::map_dfr(legacy_metrics, ~ run_tukey_one(data, .x))

kruskal_results <- purrr::map_dfr(legacy_metrics, ~ run_kruskal_one(data, .x))

dunn_results <- purrr::map_dfr(legacy_metrics, ~ run_dunn_one(data, .x))

significant_tukey <- safe_filter_sig(tukey_results, "Adjusted_P", 0.05)

significant_dunn <- safe_filter_sig(dunn_results, "Adjusted_P", 0.05)

paper_descriptive_table <- descriptive_all %>%

dplyr::mutate(

`Mean_SD` = sprintf("%.2f +/- %.2f", mean, sd),

`Median [Q1, Q3]` = sprintf("%.2f [%.2f, %.2f]", median, q1, q3),

Summary = paste0(`Mean_SD`, "; ", `Median [Q1, Q3]`)

) %>%

dplyr::select(metric_group, metric, model, Summary) %>%

tidyr::pivot_wider(names_from = model, values_from = Summary) %>%

dplyr::arrange(metric_group, metric)

paper_test_table_non_safety <- friedman_results %>%

dplyr::select(

Metric, Test, N_questions, N_models, Statistic, DF,

P_value_formatted, Kendall_W, Kendall_W_lower_95_CI,

Kendall_W_upper_95_CI, Kendall_W_95_CI, Effect_size,

Effect_size_note, Note

)

paper_test_table_safety <- cochran_q_result %>%

dplyr::transmute(

Metric, Test, N_questions, N_models, Statistic, DF,

P_value_formatted,

Kendall_W = Cochran_Q_Kendall_W,

Kendall_W_lower_95_CI = Cochran_Q_Kendall_W_lower_95_CI,

Kendall_W_upper_95_CI = Cochran_Q_Kendall_W_upper_95_CI,

Kendall_W_95_CI = Cochran_Q_Kendall_W_95_CI,

Effect_size, Effect_size_note, Note

)

paper_test_table <- dplyr::bind_rows(paper_test_table_non_safety, paper_test_table_safety)

paper_safety_table <- safety_rate %>%

dplyr::mutate(

Safety_summary = paste0(safe_n, "/", n, " (", safe_rate_percent, "%)")

) %>%

dplyr::select(model, n, safe_n, unsafe_n, safe_rate_percent, unsafe_rate_percent, Safety_summary)

readme <- data.frame(

Section = c(

"Data structure",

"Main analysis",

"Safety analysis",

"Rater agreement",

"Automatic model detection",

"Automatic rater detection",

"Legacy analysis",

"Score coding",

"Model name correction"

),

Explanation = c(

"Data should be long format: question, model, metric, score, with optional rater columns named rater1, rater2, and so on.",

"For non-safety metrics, models are compared using Friedman tests by question, with Kendall's W and bootstrap 95% CI reported as the overall effect size; pairwise paired Wilcoxon signed-rank tests include mean/median paired differences, bootstrap 95% CIs, and paired rank-biserial correlation.",

paste0("Safety is coded as ", safety_safe_value, "=safe and ", safety_unsafe_value, "=unsafe. It is summarized as safe response rate and compared using Cochran Q test with a Kendall's W-like effect size and bootstrap 95% CI; pairwise McNemar tests include paired safe-rate differences and 95% CIs."),

"Agreement is automatically calculated for Safety, Accuracy, Empathy, DISCERN, EQIP, JAMA and GQS. Safety uses Fleiss' kappa with bootstrap 95% CI; other metrics use ICC(2,1) with 95% CI.",

"Models are automatically identified from the model column. The color palette is generated automatically with redundant reserved colors.",

paste0("Detected ", rater_count, " rater column(s): ", ifelse(rater_count > 0, paste(rater_cols, collapse = "; "), "none"), "."),

"ANOVA/Tukey and Kruskal-Dunn are retained only for compatibility with the previous scripts; because the same questions are answered by all models, the paired analysis should be treated as primary.",

paste0("Accuracy and Empathy are treated as 1-5 ordinal scores. Safety is treated as a binary outcome (", safety_safe_value, "=safe, ", safety_unsafe_value, "=unsafe)."),

"Model names are automatically read from the model column without predefined model names or version correction."

)

)

wb <- createWorkbook()

add_sheet(wb, "00_README", readme)

add_sheet(wb, "01_data_check_summary", data_check_summary)

add_sheet(wb, "02_coverage", coverage)

add_sheet(wb, "03_duplicates", duplicates)

add_sheet(wb, "04_missing_combinations", missing_combinations)

add_sheet(wb, "05_score_range_check", score_range_check)

add_sheet(wb, "06_safety_rate", safety_rate)

add_sheet(wb, "07_safety_cochranQ", cochran_q_result)

add_sheet(wb, "08_safety_mcnemar", pairwise_mcnemar_results)

add_sheet(wb, "09_descriptive_all", descriptive_all)

add_sheet(wb, "10_normality", normality_results)

add_sheet(wb, "11_rater_agreement", rater_agreement_results)

add_sheet(wb, "11_main_friedman", friedman_results)

add_sheet(wb, "12_pairwise_wilcox", pairwise_wilcox_results)

add_sheet(wb, "13_sig_safety_mcnemar", significant_pairwise_mcnemar)

add_sheet(wb, "14_sig_pairwise_wilcox", significant_pairwise_wilcox)

add_sheet(wb, "15_paper_safety", paper_safety_table)

add_sheet(wb, "16_paper_descriptive", paper_descriptive_table)

add_sheet(wb, "17_paper_tests", paper_test_table)

add_sheet(wb, "18_legacy_anova", anova_results)

add_sheet(wb, "19_legacy_tukey", tukey_results)

add_sheet(wb, "20_legacy_kruskal", kruskal_results)

add_sheet(wb, "21_legacy_dunn", dunn_results)

add_sheet(wb, "22_sig_tukey", significant_tukey)

add_sheet(wb, "23_sig_dunn", significant_dunn)

excel_output <- file.path(output_dir, "LLM_full_statistical_analysis_results.xlsx")

saveWorkbook(wb, excel_output, overwrite = TRUE)

word_output <- file.path(output_dir, "LLM_paper_ready_result_tables.docx")

format_num2 <- function(x) {

ifelse(is.na(x), "", sprintf("%.2f", as.numeric(x)))

}

format_num3 <- function(x) {

ifelse(is.na(x), "", sprintf("%.3f", as.numeric(x)))

}

format_summary_cell <- function(mean, sd, median, q1, q3) {

ifelse(

is.na(mean),

"",

paste0(

sprintf("%.2f \u00B1 %.2f", mean, sd),

"; ",

sprintf("%.2f [%.2f, %.2f]", median, q1, q3)

)

)

}

make_paper_metric_table <- function(metrics_vec) {

desc_wide <- descriptive_all %>%

dplyr::filter(as.character(metric) %in% metrics_vec) %>%

dplyr::mutate(

Metric = as.character(metric),

model = as.character(model),

Summary = format_summary_cell(mean, sd, median, q1, q3)

) %>%

dplyr::select(Metric, model, Summary) %>%

tidyr::pivot_wider(names_from = model, values_from = Summary)

for (m in models_present) {

if (!m %in% names(desc_wide)) desc_wide[[m]] <- ""

}

test_part <- friedman_results %>%

dplyr::filter(Metric %in% metrics_vec) %>%

dplyr::transmute(

Metric,

`Overall P` = P_value_formatted,

`Kendall's W` = format_num3(Kendall_W),

`Kendall's W 95% CI` = dplyr::if_else(

is.na(Kendall_W_lower_95_CI) | is.na(Kendall_W_upper_95_CI),

"",

paste0(format_num3(Kendall_W_lower_95_CI), "-", format_num3(Kendall_W_upper_95_CI))

)

)

out <- desc_wide %>%

dplyr::left_join(test_part, by = "Metric") %>%

dplyr::select(Metric, dplyr::all_of(models_present), `Overall P`, `Kendall's W`, `Kendall's W 95% CI`)

out

}

make_safety_summary_table <- function() {

safety_rate %>%

dplyr::mutate(

Model = as.character(model),

`Safe responses, n (%)` = paste0(safe_n, " (", sprintf("%.1f", safe_rate_percent), ")"),

`Unsafe responses, n (%)` = paste0(unsafe_n, " (", sprintf("%.1f", unsafe_rate_percent), ")")

) %>%

dplyr::select(

Model,

N = n,

`Safe responses, n (%)`,

`Unsafe responses, n (%)`

)

}

make_safety_overall_test_table <- function() {

cochran_q_result %>%

dplyr::transmute(

Test,

`No. of questions` = N_questions,

`No. of models` = N_models,

Statistic = format_num3(Statistic),

DF,

`P value` = P_value_formatted,

`Effect size` = format_num3(Cochran_Q_Kendall_W),

`Effect size 95% CI` = dplyr::if_else(

is.na(Cochran_Q_Kendall_W_lower_95_CI) | is.na(Cochran_Q_Kendall_W_upper_95_CI),

"",

paste0(format_num3(Cochran_Q_Kendall_W_lower_95_CI), "-", format_num3(Cochran_Q_Kendall_W_upper_95_CI))

)

)

}

make_rater_agreement_table <- function() {

need_cols <- c("Metric", "Agreement_index", "Estimate", "Lower_95_CI", "Upper_95_CI", "N_complete_items", "N_raters")

if (!has_cols(rater_agreement_results, need_cols)) {

return(data.frame(Note = "No rater agreement result available."))

}

rater_agreement_results %>%

dplyr::transmute(

Metric,

`Agreement index` = Agreement_index,

Estimate = format_num3(Estimate),

`95% CI` = dplyr::if_else(

is.na(Lower_95_CI) | is.na(Upper_95_CI),

"",

paste0(format_num3(Lower_95_CI), "-", format_num3(Upper_95_CI))

),

`P value` = P_value_formatted,

`Complete items` = N_complete_items,

`No. of raters` = N_raters,

Note

)

}

make_safety_pairwise_table <- function() {

need_cols <- c(

"Model_1", "Model_2", "N_pairs", "Safe_rate_difference_percent",

"Safe_rate_difference_lower_95_CI", "Safe_rate_difference_upper_95_CI",

"Safe_to_unsafe", "Unsafe_to_safe", "Matched_OR",

"Matched_OR_lower_95_CI", "Matched_OR_upper_95_CI",

"P_adjusted_formatted", "Note"

)

if (!has_cols(pairwise_mcnemar_results, need_cols)) {

return(data.frame(Note = "No pairwise McNemar result available."))

}

out <- pairwise_mcnemar_results %>%

dplyr::transmute(

Comparison = paste(Model_1, "vs", Model_2),

`N pairs` = N_pairs,

`Safe rate difference, %` = format_num2(Safe_rate_difference_percent),

`Safe rate difference 95% CI` = dplyr::if_else(

is.na(Safe_rate_difference_lower_95_CI) | is.na(Safe_rate_difference_upper_95_CI),

"",

paste0(format_num2(Safe_rate_difference_lower_95_CI), "-", format_num2(Safe_rate_difference_upper_95_CI))

),

`Safe to unsafe, n` = Safe_to_unsafe,

`Unsafe to safe, n` = Unsafe_to_safe,

`Matched OR` = format_num2(Matched_OR),

`Matched OR 95% CI` = dplyr::if_else(

is.na(Matched_OR_lower_95_CI) | is.na(Matched_OR_upper_95_CI),

"",

paste0(format_num2(Matched_OR_lower_95_CI), "-", format_num2(Matched_OR_upper_95_CI))

),

`Adjusted P` = P_adjusted_formatted,

Note

)

if (nrow(out) == 0) out <- data.frame(Note = "No pairwise McNemar result available.")

out

}

make_significant_pairwise_table <- function() {

need_cols <- c(

"Metric", "Model_1", "Model_2", "N_pairs", "Mean_difference",

"Mean_difference_lower_95_CI", "Mean_difference_upper_95_CI",

"Median_difference", "Median_difference_lower_95_CI", "Median_difference_upper_95_CI",

"Rank_biserial_correlation", "Rank_biserial_lower_95_CI", "Rank_biserial_upper_95_CI",

"P_adjusted_formatted"

)

if (!has_cols(significant_pairwise_wilcox, need_cols)) {

return(data.frame(Note = "No significant pairwise difference after Benjamini-Hochberg correction."))

}

out <- significant_pairwise_wilcox %>%

dplyr::transmute(

Metric,

Comparison = paste(Model_1, "vs", Model_2),

`N pairs` = N_pairs,

`Mean difference` = format_num2(Mean_difference),

`Mean difference 95% CI` = dplyr::if_else(

is.na(Mean_difference_lower_95_CI) | is.na(Mean_difference_upper_95_CI),

"",

paste0(format_num2(Mean_difference_lower_95_CI), "-", format_num2(Mean_difference_upper_95_CI))

),

`Median difference` = format_num2(Median_difference),

`Median difference 95% CI` = dplyr::if_else(

is.na(Median_difference_lower_95_CI) | is.na(Median_difference_upper_95_CI),

"",

paste0(format_num2(Median_difference_lower_95_CI), "-", format_num2(Median_difference_upper_95_CI))

),

`Rank-biserial correlation` = format_num3(Rank_biserial_correlation),

`Rank-biserial 95% CI` = dplyr::if_else(

is.na(Rank_biserial_lower_95_CI) | is.na(Rank_biserial_upper_95_CI),

"",

paste0(format_num3(Rank_biserial_lower_95_CI), "-", format_num3(Rank_biserial_upper_95_CI))

),

`Adjusted P` = P_adjusted_formatted

)

if (nrow(out) == 0) out <- data.frame(Note = "No significant pairwise difference after Benjamini-Hochberg correction.")

out

}

make_significant_safety_pairwise_table <- function() {

need_cols <- c(

"Model_1", "Model_2", "N_pairs", "Safe_rate_difference_percent",

"Safe_rate_difference_lower_95_CI", "Safe_rate_difference_upper_95_CI",

"Safe_to_unsafe", "Unsafe_to_safe", "Matched_OR",

"Matched_OR_lower_95_CI", "Matched_OR_upper_95_CI",

"P_adjusted_formatted"

)

if (!has_cols(significant_pairwise_mcnemar, need_cols)) {

return(data.frame(Note = "No significant pairwise safety difference after Benjamini-Hochberg correction."))

}

out <- significant_pairwise_mcnemar %>%

dplyr::transmute(

Comparison = paste(Model_1, "vs", Model_2),

`N pairs` = N_pairs,

`Safe rate difference, %` = format_num2(Safe_rate_difference_percent),

`Safe rate difference 95% CI` = dplyr::if_else(

is.na(Safe_rate_difference_lower_95_CI) | is.na(Safe_rate_difference_upper_95_CI),

"",

paste0(format_num2(Safe_rate_difference_lower_95_CI), "-", format_num2(Safe_rate_difference_upper_95_CI))

),

`Safe to unsafe, n` = Safe_to_unsafe,

`Unsafe to safe, n` = Unsafe_to_safe,

`Matched OR` = format_num2(Matched_OR),

`Matched OR 95% CI` = dplyr::if_else(

is.na(Matched_OR_lower_95_CI) | is.na(Matched_OR_upper_95_CI),

"",

paste0(format_num2(Matched_OR_lower_95_CI), "-", format_num2(Matched_OR_upper_95_CI))

),

`Adjusted P` = P_adjusted_formatted

)

if (nrow(out) == 0) out <- data.frame(Note = "No significant pairwise safety difference after Benjamini-Hochberg correction.")

out

}

make_all_pairwise_nonbinary_table <- function() {

need_cols <- c(

"Metric", "Model_1", "Model_2", "N_pairs", "Mean_difference",

"Mean_difference_lower_95_CI", "Mean_difference_upper_95_CI",

"Median_difference", "Median_difference_lower_95_CI", "Median_difference_upper_95_CI",

"Rank_biserial_correlation", "Rank_biserial_lower_95_CI", "Rank_biserial_upper_95_CI",

"P_adjusted_formatted", "Note"

)

if (!has_cols(pairwise_wilcox_results, need_cols)) {

return(data.frame(Note = "No pairwise Wilcoxon result available."))

}

out <- pairwise_wilcox_results %>%

dplyr::transmute(

Metric,

Comparison = paste(Model_1, "vs", Model_2),

`N pairs` = N_pairs,

`Mean difference` = format_num2(Mean_difference),

`Mean difference 95% CI` = dplyr::if_else(

is.na(Mean_difference_lower_95_CI) | is.na(Mean_difference_upper_95_CI),

"",

paste0(format_num2(Mean_difference_lower_95_CI), "-", format_num2(Mean_difference_upper_95_CI))

),

`Median difference` = format_num2(Median_difference),

`Median difference 95% CI` = dplyr::if_else(

is.na(Median_difference_lower_95_CI) | is.na(Median_difference_upper_95_CI),

"",

paste0(format_num2(Median_difference_lower_95_CI), "-", format_num2(Median_difference_upper_95_CI))

),

`Rank-biserial correlation` = format_num3(Rank_biserial_correlation),

`Rank-biserial 95% CI` = dplyr::if_else(

is.na(Rank_biserial_lower_95_CI) | is.na(Rank_biserial_upper_95_CI),

"",

paste0(format_num3(Rank_biserial_lower_95_CI), "-", format_num3(Rank_biserial_upper_95_CI))

),

`Adjusted P` = P_adjusted_formatted,

Note

)

if (nrow(out) == 0) out <- data.frame(Note = "No pairwise Wilcoxon result available.")

out

}

add_word_table <- function(doc, title, df, note = NULL, font_size = 8) {

doc <- officer::body_add_par(doc, title, style = "heading 2")

ft <- flextable::flextable(df)

ft <- flextable::theme_booktabs(ft)

ft <- flextable::fontsize(ft, size = font_size, part = "all")

ft <- flextable::bold(ft, part = "header")

ft <- flextable::align(ft, align = "center", part = "all")

ft <- flextable::align(ft, j = 1, align = "left", part = "body")

ft <- flextable::valign(ft, valign = "center", part = "all")

ft <- flextable::set_table_properties(ft, layout = "autofit", width = 1)

ft <- flextable::autofit(ft)

doc <- flextable::body_add_flextable(doc, ft)

if (!is.null(note) && nchar(note) > 0) {

doc <- officer::body_add_par(doc, note, style = "Normal")

}

doc <- officer::body_add_par(doc, "", style = "Normal")

doc

}

word_table_safety <- make_safety_summary_table()

word_table_safety_test <- make_safety_overall_test_table()

word_table_rater_agreement <- make_rater_agreement_table()

word_table_accuracy_empathy <- make_paper_metric_table(c("Accuracy", "Empathy"))

word_table_reliability <- make_paper_metric_table(reliability_metrics)

word_table_readability <- make_paper_metric_table(readability_metrics)

word_table_safety_pairwise <- make_safety_pairwise_table()

word_table_sig_nonbinary <- make_significant_pairwise_table()

word_table_sig_safety <- make_significant_safety_pairwise_table()

word_table_all_pairwise_nonbinary <- make_all_pairwise_nonbinary_table()

doc <- officer::read_docx()

doc <- officer::body_add_par(doc, "Tables for Manuscript", style = "heading 1")

doc <- officer::body_add_par(

doc,

"Safety was coded as 0 = safe and 1 = unsafe. For non-binary outcomes, values are presented as mean \u00B1 standard deviation; median [interquartile range]. Overall P values for non-binary outcomes were obtained using Friedman tests, with Kendall's W reported as the effect size. Safety was compared using Cochran's Q test. Pairwise P values were adjusted using the Benjamini-Hochberg method.",

style = "Normal"

)

doc <- add_word_table(

doc,

"Table 0. Inter-rater agreement",

word_table_rater_agreement,

note = "Note: Safety was assessed using Fleiss' kappa. Accuracy, Empathy, DISCERN, EQIP, JAMA and GQS were assessed using ICC(2,1).",

font_size = 8

)

doc <- add_word_table(

doc,

"Table 1. Safety of responses across models",

word_table_safety,

note = paste0("Note: Safety was coded as ", safety_safe_value, " = safe and ", safety_unsafe_value, " = unsafe. Safe responses are presented as n (%)."),

font_size = 9

)

doc <- add_word_table(

doc,

"Table 2. Overall comparison of safety across models",

word_table_safety_test,

note = "Note: Overall comparison was performed using Cochran's Q test because Safety was a binary paired outcome. The effect size is Q/(n*(k-1)); its 95% CI was obtained by nonparametric bootstrap resampling by question.",

font_size = 9

)

doc <- add_word_table(

doc,

"Table 3. Accuracy and empathy scores across models",

word_table_accuracy_empathy,

note = "Note: Values are mean \u00B1 SD; median [Q1, Q3]. Overall P values were obtained using Friedman tests.",

font_size = 8

)

doc <- add_word_table(

doc,

"Table 4. Reliability and quality scores across models",

word_table_reliability,

note = "Note: Values are mean \u00B1 SD; median [Q1, Q3]. Overall P values were obtained using Friedman tests.",

font_size = 8

)

doc <- add_word_table(

doc,

"Table 5. Readability scores across models",

word_table_readability,

note = "Note: Values are mean \u00B1 SD; median [Q1, Q3]. For FRES, higher scores indicate easier readability; for ARI, CL, FKGL, GFI and SMOG, higher scores indicate greater reading difficulty.",

font_size = 8

)

doc <- officer::body_add_break(doc)

doc <- officer::body_add_par(doc, "Supplementary Tables", style = "heading 1")

doc <- add_word_table(

doc,

"Supplementary Table 1. Pairwise comparisons of safety",

word_table_safety_pairwise,

note = "Note: Pairwise comparisons were performed using McNemar tests with Benjamini-Hochberg correction. Safe-rate differences and their bootstrap 95% CIs are reported.",

font_size = 8

)

doc <- add_word_table(

doc,

"Supplementary Table 2. Significant pairwise comparisons for non-binary outcomes",

word_table_sig_nonbinary,

note = "Note: Pairwise comparisons were performed using paired Wilcoxon signed-rank tests with Benjamini-Hochberg correction. Paired mean/median differences and rank-biserial correlations are reported with bootstrap 95% CIs. Only significant comparisons are shown.",

font_size = 8

)

doc <- add_word_table(

doc,

"Supplementary Table 3. Significant pairwise comparisons of safety",

word_table_sig_safety,

note = "Note: Pairwise comparisons were performed using McNemar tests with Benjamini-Hochberg correction. Safe-rate differences and their bootstrap 95% CIs are reported. Only significant comparisons are shown.",

font_size = 8

)

doc <- add_word_table(

doc,

"Supplementary Table 4. All pairwise comparisons for non-binary outcomes",

word_table_all_pairwise_nonbinary,

note = "Note: Pairwise comparisons were performed using paired Wilcoxon signed-rank tests with Benjamini-Hochberg correction. Paired mean/median differences and rank-biserial correlations are reported with bootstrap 95% CIs.",

font_size = 7

)

print(doc, target = word_output)

pub_theme <- theme_minimal() +

theme(

panel.grid = element_blank(),

axis.line = element_line(color = "black", linewidth = 0.5),

axis.ticks = element_line(color = "black", linewidth = 0.5),

axis.ticks.length = unit(0.2, "cm"),

plot.title = element_text(hjust = 0.5, size = 18, face = "bold", margin = margin(b = 8)),

plot.title.position = "panel",

plot.margin = margin(12, 12, 12, 12),

axis.title = element_text(size = 16, face = "bold"),

axis.text.y = element_text(size = 14, color = "black"),

axis.text.x = element_text(size = 14, color = "black", angle = 45, hjust = 1),

legend.title = element_blank(),

legend.position = "bottom",

legend.justification = "center",

legend.box = "horizontal",

legend.direction = "horizontal",

legend.text = element_text(size = 12)

)

mean_scores <- data %>%

dplyr::filter(metric != "Safety") %>%

dplyr::group_by(metric_group, metric, model) %>%

dplyr::summarise(mean_score = round(mean(score, na.rm = TRUE), 2), .groups = "drop")

safety_plot_data <- safety_rate %>%

dplyr::mutate(

metric = "Safety",

metric_group = "1. Safety",

mean_score = safe_rate_percent

)

get_y_settings <- function(metric_name) {

if (metric_name %in% c("DISCERN", "EQIP", "FRES")) {

return(list(limits = c(0, 100), breaks = seq(0, 100, 20), ref = numeric(0), ylab = "Mean Score"))

}

if (metric_name %in% c("ARI", "CL", "FKGL", "GFI", "SMOG")) {

return(list(limits = c(0, 20), breaks = seq(0, 20, 5), ref = 6, ylab = "Mean Score"))

}

if (metric_name %in% c("GQS", "JAMA", "Accuracy", "Empathy")) {

return(list(limits = c(0, 5), breaks = seq(0, 5, 1), ref = numeric(0), ylab = "Mean Score"))

}

if (metric_name == "Safety") {

return(list(limits = c(0, 100), breaks = seq(0, 100, 20), ref = numeric(0), ylab = "Safe responses (%)"))

}

list(limits = NULL, breaks = waiver(), ref = numeric(0), ylab = "Score")

}

make_metric_bar <- function(metric_name) {

ys <- get_y_settings(metric_name)

if (metric_name == "Safety") {

plot_df <- safety_plot_data

y_var <- "mean_score"

label_text <- "mean_score"

} else {

plot_df <- mean_scores %>% dplyr::filter(metric == metric_name)

y_var <- "mean_score"

label_text <- "mean_score"

}

if (nrow(plot_df) == 0) return(NULL)

p <- ggplot(plot_df, aes(x = model, y = .data[[y_var]], fill = model)) +

geom_col(width = 0.7) +

geom_text(

aes(label = sprintf("%.2f", .data[[label_text]])),

vjust = -0.3, size = 5, fontface = "bold"

) +

scale_fill_manual(values = sci_colors, drop = FALSE) +

guides(

fill = guide_legend(

nrow = 1,

byrow = TRUE,

override.aes = list(size = 5)

)

) +

labs(title = metric_name, x = NULL, y = ys$ylab) +

pub_theme

if (!is.null(ys$limits)) {

p <- p + scale_y_continuous(

limits = ys$limits,

breaks = ys$breaks,

expand = expansion(mult = c(0, 0.05))

)

}

if (length(ys$ref) > 0) {

p <- p + geom_hline(

yintercept = ys$ref,

color = "red",

linetype = "dashed",

linewidth = 1

)

}

p

}

plot_objects <- list()

plot_metrics <- c("Safety", "Accuracy", "Empathy", reliability_metrics, readability_metrics)

for (m in plot_metrics) {

p <- make_metric_bar(m)

if (!is.null(p)) {

plot_objects[[m]] <- p

}

}

save_combined_plot <- function(metrics, filename, ncol, width, height) {

ps <- plot_objects[metrics]

ps <- ps[!vapply(ps, is.null, logical(1))]

if (length(ps) == 0) return(NULL)

combined <- patchwork::wrap_plots(

plotlist = ps,

ncol = ncol,

guides = "collect"

) &

theme(

legend.position = "bottom",

legend.justification = "center",

legend.box = "horizontal",

legend.direction = "horizontal",

legend.title = element_blank(),

legend.text = element_text(size = 12),

legend.margin = margin(t = 5, r = 0, b = 5, l = 0),

legend.box.margin = margin(t = 5, r = 0, b = 5, l = 0)

)

ggsave(

filename = file.path(plot_dir, filename),

plot = combined,

width = width,

height = height,

units = "in",

dpi = 600,

compression = "lzw",

bg = "white"

)

combined

}

combined_performance <- save_combined_plot(

c("Safety", "Accuracy", "Empathy"),

"Figure1_combined_safety_accuracy_empathy.tif",

ncol = 3,

width = 15,

height = 6.5

)

combined_reliability <- save_combined_plot(

reliability_metrics,

"Figure2_combined_reliability_quality.tif",

ncol = 4,

width = 20,

height = 6.5

)

combined_readability <- save_combined_plot(

readability_metrics,

"Figure3_combined_readability.tif",

ncol = 6,

width = 30,

height = 6.5

)

paired_distribution_dir <- file.path(plot_dir, "paired_distribution_figures")

if (!dir.exists(paired_distribution_dir)) dir.create(paired_distribution_dir, recursive = TRUE)

dist_theme <- theme_minimal() +

theme(

panel.grid.minor = element_blank(),

panel.grid.major.x = element_blank(),

axis.line = element_line(color = "black", linewidth = 0.5),

axis.ticks = element_line(color = "black", linewidth = 0.4),

strip.text = element_text(size = 13, face = "bold"),

axis.title = element_text(size = 14, face = "bold"),

axis.text.x = element_text(size = 11, color = "black", angle = 45, hjust = 1),

axis.text.y = element_text(size = 11, color = "black"),

legend.title = element_blank(),

legend.text = element_text(size = 11),

plot.title = element_text(size = 16, face = "bold", hjust = 0.5),

plot.margin = margin(10, 10, 10, 10)

)

make_box_jitter_distribution <- function(metrics_vec, title_text) {

plot_df <- data %>%

dplyr::filter(as.character(metric) %in% metrics_vec, metric != "Safety") %>%

dplyr::mutate(

metric = factor(as.character(metric), levels = metrics_vec),

model = factor(as.character(model), levels = models_present)

)

if (nrow(plot_df) == 0) return(NULL)

p <- ggplot(plot_df, aes(x = model, y = score, fill = model, color = model)) +

geom_boxplot(

width = 0.55,

alpha = 0.45,

outlier.shape = NA,

linewidth = 0.55,

show.legend = TRUE,

key_glyph = "rect"

) +

geom_jitter(

width = 0.14,

alpha = 0.60,

size = 1.7,

show.legend = FALSE

) +

stat_summary(

fun = median,

geom = "point",

shape = 23,

size = 2.3,

fill = "white",

color = "black",

show.legend = FALSE

) +

scale_fill_manual(values = sci_colors, drop = FALSE, name = NULL) +

scale_color_manual(values = sci_colors, drop = FALSE, guide = "none") +

facet_wrap(~ metric, scales = "free_y", nrow = 1) +

labs(

title = title_text,

x = NULL,

y = "Score"

) +

guides(

fill = guide_legend(

nrow = 1,

byrow = TRUE,

override.aes = list(

shape = 22,

size = 3.2,

alpha = 1,

color = NA,

linetype = 0

)

)

) +

dist_theme +

theme(

legend.position = "bottom",

legend.direction = "horizontal",

legend.justification = "center",

legend.box = "horizontal",

legend.key.width = unit(0.45, "cm"),

legend.key.height = unit(0.30, "cm"),

legend.text = element_text(size = 10),

legend.spacing.x = unit(0.15, "cm")

)

ref_metrics <- intersect(metrics_vec, readability_metrics)

if (length(ref_metrics) > 0) {

ref_df <- data.frame(

metric = factor(ref_metrics, levels = metrics_vec),

yintercept = ifelse(ref_metrics == "FRES", 80, 6)

)

p <- p +

geom_hline(

data = ref_df,

aes(yintercept = yintercept),

inherit.aes = FALSE,

linetype = "dashed",

linewidth = 0.7,

color = "red"

)

}

p

}

make_safety_composition_plot <- function() {

if (!"Safety" %in% unique(as.character(data$metric))) return(NULL)

safety_distribution_df <- data %>%

dplyr::filter(as.character(metric) == "Safety") %>%

dplyr::mutate(

model = factor(as.character(model), levels = models_present),

score = as.numeric(score),

Safety_status = dplyr::case_when(

score == safety_unsafe_value ~ "Unsafe",

score == safety_safe_value ~ "Safe",

TRUE ~ NA_character_

),

Safety_status = factor(Safety_status, levels = c("Unsafe", "Safe"))

) %>%

dplyr::filter(!is.na(Safety_status)) %>%

dplyr::count(model, Safety_status, name = "n") %>%

tidyr::complete(

model = factor(models_present, levels = models_present),

Safety_status = factor(c("Unsafe", "Safe"), levels = c("Unsafe", "Safe")),

fill = list(n = 0)

) %>%

dplyr::group_by(model) %>%

dplyr::mutate(

total_n = sum(n, na.rm = TRUE),

percent = dplyr::if_else(total_n > 0, 100 * n / total_n, 0),

label = dplyr::if_else(

n > 0,

paste0(n, " (", sprintf("%.1f", percent), "%)"),

""

)

) %>%

dplyr::ungroup()

p <- ggplot(

safety_distribution_df,

aes(x = model, y = percent, fill = Safety_status)

) +

geom_col(width = 0.65, color = "white", linewidth = 0.4) +

geom_text(

aes(label = label),

position = position_stack(vjust = 0.5),

size = 3.0,

fontface = "bold"

) +

scale_fill_manual(

values = c("Unsafe" = "#D73027", "Safe" = "#1A9850"),

breaks = c("Unsafe", "Safe"),

drop = FALSE

) +

scale_y_continuous(

limits = c(0, 100),

breaks = seq(0, 100, 20),

expand = expansion(mult = c(0, 0.02))

) +

labs(

title = "Safety",

x = NULL,

y = "Percentage of responses (%)"

) +

guides(

fill = guide_legend(

nrow = 1,

byrow = TRUE,

override.aes = list(

shape = 22,

size = 3.2,

alpha = 1,

color = NA,

linetype = 0

)

)

) +

dist_theme +

theme(

legend.position = "bottom",

legend.direction = "horizontal",

legend.key.width = unit(0.45, "cm"),

legend.key.height = unit(0.30, "cm"),

legend.text = element_text(size = 10),

legend.spacing.x = unit(0.15, "cm")

)

p

}

safety_dist_plot <- make_safety_composition_plot()

accuracy_empathy_dist_plot <- make_box_jitter_distribution(

metrics_vec = c("Accuracy", "Empathy"),

title_text = "Accuracy and Empathy"

)

figure1_distribution_list <- list(safety_dist_plot, accuracy_empathy_dist_plot)

figure1_distribution_list <- figure1_distribution_list[!vapply(figure1_distribution_list, is.null, logical(1))]

if (length(figure1_distribution_list) > 0) {

figure1_distribution <- patchwork::wrap_plots(

plotlist = figure1_distribution_list,

nrow = 1

) +

patchwork::plot_annotation(

title = "Distribution of Safety, Accuracy and Empathy Across Models"

) &

theme(

plot.title = element_text(size = 18, face = "bold", hjust = 0.5)

)

ggsave(

filename = file.path(paired_distribution_dir, "Figure1_distribution_safety_accuracy_empathy.tif"),

plot = figure1_distribution,

width = 16,

height = 6.5,

units = "in",

dpi = 600,

compression = "lzw",

bg = "white"

)

}

figure2_distribution <- make_box_jitter_distribution(

metrics_vec = reliability_metrics,

title_text = "Distribution of Reliability and Quality Scores Across Models"

)

if (!is.null(figure2_distribution)) {

ggsave(

filename = file.path(paired_distribution_dir, "Figure2_distribution_reliability_quality.tif"),

plot = figure2_distribution,

width = 20,

height = 6.5,

units = "in",

dpi = 600,

compression = "lzw",

bg = "white"

)

}

figure3_distribution <- make_box_jitter_distribution(

metrics_vec = readability_metrics,

title_text = "Distribution of Readability Scores Across Models"

)

if (!is.null(figure3_distribution)) {

ggsave(

filename = file.path(paired_distribution_dir, "Figure3_distribution_readability.tif"),

plot = figure3_distribution,

width = 30,

height = 6.5,

units = "in",

dpi = 600,

compression = "lzw",

bg = "white"

)

}

cat("\n==========================================================\n")

cat("Analysis completed.\n")

cat("Excel output: ", excel_output, "\n")

cat("Word output: ", word_output, "\n")

cat("Plot directory: ", plot_dir, "\n")

cat("Distribution plot directory: ", paired_distribution_dir, "\n")

cat("Three horizontal combined bar figures are exported once: Figure1, Figure2, and Figure3; each figure has one shared legend centered below.\n")

cat("Three corresponding distribution figures are exported: Figure1_distribution_safety_accuracy_empathy.tif, Figure2_distribution_reliability_quality.tif, and Figure3_distribution_readability.tif.\n")

cat("Main analysis: non-Safety metrics use Friedman tests and paired Wilcoxon tests; Safety uses Cochran Q tests and McNemar tests.\n")

cat("Result and figure order: Safety -> Accuracy -> Empathy -> Reliability -> Readability.\n")

cat("Safety coding: ", safety_safe_value, "=safe, ", safety_unsafe_value, "=unsafe.\n", sep = "")

cat("Detected models: ", paste(models_present, collapse = "; "), "\n")

cat("Detected rater columns: ", ifelse(rater_count > 0, paste(rater_cols, collapse = "; "), "No rater columns detected"), "\n")

cat("==========================================================\n\n")

cat("Data integrity summary:\n")

print(data_check_summary)

cat("\nMain analysis results:\n")

print(friedman_results)

cat("\nSafety rate:\n")

print(safety_rate)

cat("\nSafety Cochran Q:\n")

print(cochran_q_result)

cat("\nRater agreement:\n")

print(rater_agreement_results)
