# Supplementary material for: Safety, accuracy, empathy, reliability, and readability of large language model chatbot responses to public-facing vegetarian and vegan nutrition advice questions: a cross-sectional comparative study
Source: Front Public Health. 2026 Aug 3;14:1921819. doi: 10.3389/fpubh.2026.1921819 (PMC13478225; doi:10.3389/fpubh.2026.1921819)
Supplement: Supplementary file 6 [file Table_1.docx]

**Supplementary Table S1.CHART checklist**

| Heading | No | Chart checklist item | Page No |
| --- | --- | --- | --- |
| Title and abstract |  |  |  |
| Title | 1a | State that the study is assessing one or more generative AI-driven chatbots for clinical evidence or health advice. | Title: title identifies evaluation of LLM chatbot responses to public-facing vegetarian/vegan nutrition advice. |
| Abstract/summary | 1b | Apply a structured format, if applicable. | Abstract: structured abstract. |
| Introduction |  |  |  |
| Background | 2a | State the scientific background, rationale, and healthcare context for evaluating the generative AI-driven chatbot(s), referencing relevant literature when applicable. | Introduction: background, rationale, and health/nutrition context. |
|  | 2b | State the aims and research questions including the target audience, intervention, comparator(s), and outcome(s). | Introduction: aims, target public users, five chatbot comparators, and outcomes. |
| Methods |  |  |  |
| Model identifiers | 3a | State the name and version identifier(s) of the generative AI model(s) and chatbot(s) under evaluation, as well as their date of release or last update. | Methods; Supplementary Table S2: chatbot names, user-visible identifiers/versions, modes, query dates/location, and backend notes. |
|  | 3b | State whether the generative AI model(s) and chatbot(s) are open-source or closed-source/proprietary. | Methods; Supplementary Table S2: source availability and proprietary/hosted status. |
| Model details | 4a | State whether the generative AI model was a base model or a novel base model, tuned model, or fine-tuned model. | Methods; Supplementary Table S2: deployed web-based products/interface configurations, not local base models. |
|  | 4b | If a base model is used, cite its development in sufficient detail to identify the model. | Methods; Supplementary Table S2: user-visible model details reported where available; undisclosed backend details noted. |
|  | 4c | If a novel base model, tuned model, or fine-tuned model is used, describe the pre- and/or post-implementation/deployment data and parameters. | Methods; Supplementary Table S2: no API, local deployment, custom fine-tuning, investigator-developed RAG, or additional deployment; backend parameters unavailable. |
| Prompt engineering | 5a | Describe the evolution of study prompt development. | Methods; Supplementary File S1: question pool, screening decisions, final selection, and wording refinement. |
|  | 5ai | Describe the sources of prompts. | Methods; Supplementary File S1: prompt/question sources and screening record. |
|  | 5aii | State the number and characteristics of the individual(s) involved in prompt engineering. | Methods; Supplementary File S1: JZ/CL generated questions, JZ/QZ screened them, and 10 lay representatives reviewed wording. |
|  | 5aiii | Provide details of any patient and public involvement during prompt engineering. | Methods; Supplementary File S1: lay public involvement limited to wording refinement; no role in evidence mapping, querying, evaluation, analysis, or interpretation. |
|  | 5b | Provide study prompts. | Methods; Supplementary File S3: final prompts were question text only; complete prompt set and verbatim responses provided. |
| Query strategy | 6a | State route of access to generative AI model. | Methods; Supplementary Table S2: official web-based chatbot interfaces. |
|  | 6b | State the date(s) and location(s) of queries for the generative AI-driven chatbot(s) including the day, month, and year as well as city and country. | Methods; Supplementary Table S2: queries conducted 20-27 May 2026 in Chongqing, China. |
|  | 6c | Describe whether prompts were input into separate chat session(s). | Methods; Supplementary Table S2: single-turn, separate-session format; visible history cleared; memory/personalization disabled where available. |
|  | 6d | Provide all generative AI-driven chatbot output/responses | Methods; Supplementary File S3: complete verbatim plain-text responses provided. |
| Performance evaluation | 7a | Define the ground truth or reference standard used to define successful generative AI-driven chatbot performance. | Methods; Supplementary File S2: predefined reference-answer anchors and question-evidence mapping. |
|  | 7b | Describe the process undertaken for generative AI-driven chatbot performance evaluation. | Methods; Supplementary Table S3; Supplementary File S4: scoring instruments, blinded rating workflow, aggregation rules, and qualitative safety review. |
|  | 7bi | State the number and characteristics of team members involved in performance evaluation. | Methods; Supplementary Table S3: five blinded raters with clinical nutrition training and >=2 years of experience. |
|  | 7bii | Provide details of any patients and public involvement during the evaluation process. | Methods; Supplementary Table S3: lay public involved only in wording refinement; no patients/public in response evaluation. |
|  | 7biii | State whether evaluators were blinded to the identity of the generative AI-driven chatbot(s) under assessment. | Methods; Supplementary Table S3: model names and chatbot-specific identifiers removed before rating. |
| Sample size | 8 | Report how the sample size was determined. | Methods; Supplementary File S1; Supplementary File S5: 58 questions x 5 chatbots = 290 responses; no formal sample-size calculation. |
| Data analysis | 9a | Describe statistical analysis methods, including any evaluation of reproducibility of generative AI-driven chatbot responses. | Methods; Discussion; Supplementary Files S5-S7; Supplementary Tables S5-S9: paired statistical methods and code; within-model variability and temporal stability were not evaluated; response reproducibility was not assessed and is stated in limitations. |
|  | 9ai | Report the measures used for performance evaluation. | Methods; Supplementary Tables S3-S4: safety, accuracy, empathy, DISCERN, EQIP, JAMA, GQS, and readability measures with formulas. |
| Results |  |  |  |
|  | 10a | Report the performance evaluation undertaken including the alignment between generative AI-driven chatbot output and ground truth or reference standard using quantitative or mixed methods approaches as applicable. | Results; Tables 1-3; Figures 1-4; Supplementary Tables S5-S9; Supplementary Files S5-S6: performance and readability results/datasets. |
|  | 10b | For responses deviating from the ground truth or reference standard, state the nature of the difference(s). | Results; Supplementary File S4: deviations and risk patterns for safety-flagged responses. |
|  | 10c | Report the evaluation for potentially harmful, biased, or misleading responses. | Results; Supplementary File S4: potentially harmful/misleading responses evaluated; no separate bias analysis. |
| Discussion |  |  |  |
|  | 11a | Interpret study findings in the context of relevant evidence. | Discussion: interpretation in context of relevant vegetarian/vegan nutrition and LLM evidence. |
|  | 11b | Describe the strengths and limitations of the study. | Discussion: strengths and limitations. |
|  | 11c | Describe the potential implications for practice, education, policy, regulation, and research. | Discussion: implications for public users, clinicians/dietitians, developers, and researchers. |
| Open science |  |  |  |
| Disclosures | 12a | Report any relevant conflicts of interest for all authors. | Conflict of interest: no competing interests. |
| Funding | 12b | Report sources of funding and their role in the conduct and reporting of the study. | Funding: no specific funding. |
| Ethics | 12c | Describe the process undertaken for ethical approval. | Methods; Ethics statement: ethics approval and informed consent not required. |
|  | 12ci | Describe the measures taken to safeguard data privacy of patient health information, as applicable. | Methods; Ethics statement: no patients, medical records, identifiable personal information, or individual-level health data collected. |
|  | 12cii | State whether permission/licensing was obtained for the use of original, copyrighted data. | Methods; Ethics statement; Supplementary File S1: public sources used for theme identification; no usernames, forum posts, or identifiable/copyrighted user content collected or reproduced. |
| Protocol | 12d | Provide a study protocol. | Methods: study procedures and query protocol described; no separate registered protocol stated. |
| Data availability | 12e | State where study data, code repository, and model parameters can be accessed. | Methods; Data availability statement; Supplementary Files S2-S7; Supplementary Table S2: data/code/supplementary materials available; backend model parameters unavailable/not disclosed. |

Note: AI, artificial intelligence.
