# Supplementary material for: Safety, accuracy, empathy, reliability, and readability of large language model chatbot responses to public-facing vegetarian and vegan nutrition advice questions: a cross-sectional comparative study
Source: Front Public Health. 2026 Aug 3;14:1921819. doi: 10.3389/fpubh.2026.1921819 (PMC13478225; doi:10.3389/fpubh.2026.1921819)
Supplement: Supplementary file 7 [file Table_2.docx]

**Supplementary Table S2. Model identifiers, access conditions, and query settings**

| **Chatbot product** | **Provider** | **Access route** | **Source availability/status of the evaluated product** | **User-visible model or interface identifier recorded at query time** | **Selected response mode or configuration** | **Query dates and location** | **Release/last-update or backend details available to investigators** |
| --- | --- | --- | --- | --- | --- | --- | --- |
| ChatGPT | OpenAI | Official web-based chatbot interface | Proprietary deployed web-based chatbot product | GPT-5.4 Thinking / Thinking mode, as displayed or available at query time | Standard mode | 20 May 2026 to 27 May 2026; Chongqing, China | GPT-5.4 Thinking was publicly documented in ChatGPT before the query period; complete backend routing and deployment details for each response were not disclosed in the user-facing interface. |
| Gemini | Google | Official web-based chatbot interface | Proprietary deployed web-based chatbot product | Gemini web interface; complete backend model identifier not disclosed in the user-facing interface; Gemini 3.5 Flash was publicly documented as available during the query period | Think mode enabled | 20 May 2026 to 27 May 2026; Chongqing, China | Gemini 3.5 Flash was publicly documented as available during the query period; complete backend routing and deployment details for each response were not disclosed in the user-facing interface. |
| Microsoft Copilot | Microsoft | Official web-based chatbot interface | Proprietary deployed web-based chatbot product | Copilot web interface; complete backend model identifier not disclosed in the user-facing interface | Think Deeper mode | 20 May 2026 to 27 May 2026; Chongqing, China | Think Deeper was available as a user-facing Copilot mode; release or last-update information for the backend model used for each response was not disclosed in the user-facing interface. |
| DeepSeek | DeepSeek | Official web-based chatbot interface | Publicly accessible hosted web-based chatbot product; underlying DeepSeek-V4 Preview model was open-sourced, but complete web deployment parameters were not disclosed in the user-facing interface | DeepSeek-V4-Pro, as displayed in the web interface | Expert mode, Deep Thinking, and Intelligent Search enabled | 20 May 2026 to 27 May 2026; Chongqing, China | DeepSeek-V4 Preview, including DeepSeek-V4-Pro, was publicly documented as live before the query period; training data, backend routing, and complete deployment parameters for the hosted web chatbot were not disclosed in the user-facing interface. |
| Doubao | ByteDance / Doubao | Official web-based chatbot interface | Proprietary deployed web-based chatbot product | Seed-Doubao V3.7.2, as displayed in the user-facing interface; complete backend model identifier not disclosed | Thinking mode enabled | 20 May 2026 to 27 May 2026; Chongqing, China | The user-facing interface label was recorded by the investigators; release or last-update information, backend routing, and complete deployment details for each response were not disclosed in the user-facing interface. |

**Note:** All chatbot systems were queried through their official web-based interfaces from Chongqing, China, between 20 May 2026 and 27 May 2026. This table reports product-level identifiers, user-visible model or interface labels, and selected response modes available to or selected by the investigators at the time of querying. The study did not use application programming interface access, local deployment, investigator-developed retrieval-augmented generation, or custom fine-tuning. Each prompt was submitted once in English using a single-turn, separate-session procedure. After each response was saved, the corresponding prompt-response pair was deleted from the visible conversation history, and a new chat session was created before the next question was submitted. History-based memory or personalization was disabled where available, and no follow-up prompts were used. Built-in product-level features selected within the official interfaces, such as DeepSeek Intelligent Search, were treated as part of the user-facing chatbot configuration and recorded accordingly. When release dates, last-update dates, backend routing, complete underlying model identifiers, training data, or deployment parameters were not disclosed in the user-facing interface or could not be reliably verified by the investigators, these items were recorded as not available rather than inferred from secondary or unofficial sources.
