# Supplementary material for: Safety, accuracy, empathy, reliability, and readability of large language model chatbot responses to public-facing vegetarian and vegan nutrition advice questions: a cross-sectional comparative study
Source: Front Public Health. 2026 Aug 3;14:1921819. doi: 10.3389/fpubh.2026.1921819 (PMC13478225; doi:10.3389/fpubh.2026.1921819)
Supplement: Supplementary file 9 [file Table_4.docx]

**Supplementary Table S4. Readability metrics and formulas**

| **Metric** | **Formula used in the study** | **Required text variables** | **Interpretation** |
| --- | --- | --- | --- |
| Automated Readability Index (ARI) | ARI = 4.71 x (characters / words) + 0.5 x (words / sentences) - 21.43 | Characters, words, and sentences. | Lower values indicate easier readability and approximate a lower reading grade level. |
| Coleman-Liau Index (CL) | CLI = 0.0588 x [(letters / words) x 100] - 0.296 x [(sentences / words) x 100] - 15.8 | Letters, words, and sentences. | Lower values indicate easier readability and approximate a lower reading grade level. |
| Flesch-Kincaid Grade Level (FKGL) | FKGL = 0.39 x (words / sentences) + 11.8 x (syllables / words) - 15.59 | Words, sentences, and syllables. | Lower values indicate easier readability and approximate a lower reading grade level. |
| Gunning Fog Index (GFI) | GFI = 0.4 x [(words / (sentences + compound sentences)) + 100 x (FOG hard words / words)] | Words, sentences, compound sentences, and FOG hard words. | Lower values indicate easier readability and approximate a lower reading grade level. |
| Simple Measure of Gobbledygook (SMOG) | SMOG = 1.043 x sqrt [hard words x (30 / sentences) + 3.1291] | Hard words and sentences. | Lower values indicate easier readability and approximate a lower reading grade level. |
| Flesch Reading Ease Score (FRES) | FRES = 206.835 - 1.015 x (words / sentences) - 84.6 x (syllables / words) | Words, sentences, and syllables. | Higher values indicate easier readability. |

Abbreviations: ARI, Automated Readability Index; CL, Coleman-Liau Index; FKGL, Flesch-Kincaid Grade Level; GFI, Gunning Fog Index; SMOG, Simple Measure of Gobbledygook; FRES, Flesch Reading Ease Score. These formula-based readability metrics were applied only to complete English-language chatbot responses. They were not applied to non-English or substantially mixed-language text.
