# Supplementary material for: Safety, accuracy, empathy, reliability, and readability of large language model chatbot responses to public-facing vegetarian and vegan nutrition advice questions: a cross-sectional comparative study
Source: Front Public Health. 2026 Aug 3;14:1921819. doi: 10.3389/fpubh.2026.1921819 (PMC13478225; doi:10.3389/fpubh.2026.1921819)
Supplement: Supplementary file 10 [file Table_5.docx]

**Supplementary Table S5. Inter-rater agreement for human-rated outcomes**

| Metric | Agreement index | Estimate | 95% CI | *P* value | Complete items | No. of raters | Note |
| --- | --- | --- | --- | --- | --- | --- | --- |
| Safety | Fleiss' *kappa* | 0.823 | 0.762-0.870 | <0.001 | 290 | 5 | Primary agreement index for Safety; 95% CI based on 3000 nonparametric bootstrap resamples. A negative lower CI may occur when unsafe outcomes are extremely sparse because kappa can theoretically be below 0; this does not by itself indicate a coding error. |
| Accuracy | ICC(2,1) | 0.875 | 0.854-0.894 | <0.001 | 290 | 5 | Two-way random-effects, absolute-agreement, single-measure ICC. |
| Empathy | ICC(2,1) | 0.882 | 0.862-0.901 | <0.001 | 290 | 5 | Two-way random-effects, absolute-agreement, single-measure ICC. |
| DISCERN | ICC(2,1) | 0.877 | 0.856-0.896 | <0.001 | 290 | 5 | Two-way random-effects, absolute-agreement, single-measure ICC. |
| EQIP | ICC(2,1) | 0.878 | 0.857-0.897 | <0.001 | 290 | 5 | Two-way random-effects, absolute-agreement, single-measure ICC. |
| JAMA | ICC(2,1) | 0.872 | 0.850-0.892 | <0.001 | 290 | 5 | Two-way random-effects, absolute-agreement, single-measure ICC. |
| GQS | ICC(2,1) | 0.876 | 0.855-0.895 | <0.001 | 290 | 5 | Two-way random-effects, absolute-agreement, single-measure ICC. |

Note: Safety was assessed using Fleiss' *kappa*. Accuracy, Empathy, DISCERN, EQIP, JAMA and GQS were assessed using ICC(2,1).
