# Supplementary material for: Safety, accuracy, empathy, reliability, and readability of large language model chatbot responses to public-facing vegetarian and vegan nutrition advice questions: a cross-sectional comparative study
Source: Front Public Health. 2026 Aug 3;14:1921819. doi: 10.3389/fpubh.2026.1921819 (PMC13478225; doi:10.3389/fpubh.2026.1921819)
Supplement: Supplementary file 11 [file Table_6.docx]

**Supplementary Table S6. Pairwise comparisons of safety**

| **Comparison** | **N pairs** | **Safe rate difference, %** | **Safe rate difference 95% CI** | **Safe to unsafe, n** | **Unsafe to safe, n** | **Matched OR** | **Matched OR 95% CI** | **Adjusted *P*** | **Note** |
| --- | --- | --- | --- | --- | --- | --- | --- | --- | --- |
| ChatGPT vs Gemini | 58 | 8.62 | 1.72-17.24 | 5 | 0 | 11.00 | 0.61-198.94 | 0.147 | Pairwise McNemar test |
| ChatGPT vs Copilot | 58 | 1.72 | 0.00-5.17 | 1 | 0 | 3.00 | 0.12-73.65 | 1.000 | Pairwise McNemar test |
| ChatGPT vs DeepSeek | 58 | -1.72 | -6.90-3.45 | 1 | 2 | 0.50 | 0.05-5.51 | 1.000 | Pairwise McNemar test |
| ChatGPT vs Doubao | 58 | 12.07 | 5.17-20.69 | 7 | 0 | 15.00 | 0.86-262.65 | 0.103 | Pairwise McNemar test |
| Gemini vs Copilot | 58 | -6.90 | -13.79--1.72 | 0 | 4 | 0.11 | 0.01-2.06 | 0.223 | Pairwise McNemar test |
| Gemini vs DeepSeek | 58 | -10.34 | -18.97--3.45 | 0 | 6 | 0.08 | 0.00-1.37 | 0.103 | Pairwise McNemar test |
| Gemini vs Doubao | 58 | 3.45 | 0.00-8.62 | 2 | 0 | 5.00 | 0.24-104.15 | 0.685 | Pairwise McNemar test |
| Copilot vs DeepSeek | 58 | -3.45 | -10.34-3.45 | 1 | 3 | 0.33 | 0.03-3.20 | 0.771 | Pairwise McNemar test |
| Copilot vs Doubao | 58 | 10.34 | 3.45-18.97 | 6 | 0 | 13.00 | 0.73-230.77 | 0.103 | Pairwise McNemar test |
| DeepSeek vs Doubao | 58 | 13.79 | 5.17-22.41 | 8 | 0 | 17.00 | 0.98-294.55 | 0.103 | Pairwise McNemar test |

Note: Pairwise comparisons were performed using McNemar tests with Benjamini-Hochberg correction. Safe-rate differences and their bootstrap 95% CIs are reported.
