# Supplementary material for: Safety, accuracy, empathy, reliability, and readability of large language model chatbot responses to public-facing vegetarian and vegan nutrition advice questions: a cross-sectional comparative study
Source: Front Public Health. 2026 Aug 3;14:1921819. doi: 10.3389/fpubh.2026.1921819 (PMC13478225; doi:10.3389/fpubh.2026.1921819)
Supplement: Supplementary file 12 [file Table_7.docx]

**Supplementary Table S7. Pairwise post hoc comparisons showing statistically significant differences in accuracy and empathy.**

| **Metric** | **Comparison** | **N pairs** | **Mean difference** | **Mean difference 95% CI** | **Median difference** | **Median difference 95% CI** | **Rank-biserial correlation** | **Rank-biserial 95% CI** | **Adjusted *P*** |
| --- | --- | --- | --- | --- | --- | --- | --- | --- | --- |
| Accuracy | ChatGPT vs Gemini | 58 | 0.41 | 0.26-0.55 | 0.10 | 0.00-0.80 | 0.821 | 0.613-0.957 | <0.001 |
| Accuracy | ChatGPT vs Doubao | 58 | 0.53 | 0.37-0.69 | 0.80 | 0.10-1.00 | 0.822 | 0.640-0.956 | <0.001 |
| Accuracy | Gemini vs Copilot | 58 | -0.56 | -0.72--0.40 | -0.20 | -0.80--0.10 | -0.870 | -0.962--0.719 | <0.001 |
| Accuracy | Gemini vs DeepSeek | 58 | -0.25 | -0.41--0.08 | 0.00 | -0.20-0.00 | -0.494 | -0.760--0.183 | 0.008 |
| Accuracy | Copilot vs DeepSeek | 58 | 0.31 | 0.12-0.51 | 0.00 | 0.00-0.20 | 0.545 | 0.223-0.800 | 0.006 |
| Accuracy | Copilot vs Doubao | 58 | 0.68 | 0.52-0.85 | 0.80 | 0.20-1.00 | 0.950 | 0.862-0.993 | <0.001 |
| Accuracy | DeepSeek vs Doubao | 58 | 0.38 | 0.19-0.56 | 0.20 | 0.00-0.90 | 0.571 | 0.244-0.844 | 0.002 |
| Empathy | ChatGPT vs Gemini | 58 | 0.43 | 0.23-0.62 | 0.30 | 0.00-0.80 | 0.627 | 0.335-0.856 | <0.001 |
| Empathy | ChatGPT vs Copilot | 58 | 0.44 | 0.25-0.65 | 0.20 | 0.00-0.80 | 0.669 | 0.387-0.889 | <0.001 |
| Empathy | ChatGPT vs DeepSeek | 58 | 0.52 | 0.33-0.71 | 0.50 | 0.00-1.00 | 0.749 | 0.525-0.911 | <0.001 |
| Empathy | ChatGPT vs Doubao | 58 | 0.65 | 0.42-0.88 | 0.80 | 0.20-1.00 | 0.739 | 0.529-0.911 | <0.001 |

Note: Pairwise comparisons were performed using paired Wilcoxon signed-rank tests with Benjamini-Hochberg correction. Paired mean/median differences and rank-biserial correlations are reported with bootstrap 95% CIs. Only significant comparisons are shown.
