# Supplementary material for: Safety, accuracy, empathy, reliability, and readability of large language model chatbot responses to public-facing vegetarian and vegan nutrition advice questions: a cross-sectional comparative study
Source: Front Public Health. 2026 Aug 3;14:1921819. doi: 10.3389/fpubh.2026.1921819 (PMC13478225; doi:10.3389/fpubh.2026.1921819)
Supplement: Supplementary file 13 [file Table_8.docx]

**Supplementary Table S8. Pairwise post hoc comparisons showing statistically significant differences in reliability and information-quality metrics**

| **Metric** | **Comparison** | **N pairs** | **Mean difference** | **Mean difference 95% CI** | **Median difference** | **Median difference 95% CI** | **Rank-biserial correlation** | **Rank-biserial 95% CI** | **Adjusted *P*** |
| --- | --- | --- | --- | --- | --- | --- | --- | --- | --- |
| DISCERN | ChatGPT vs Gemini | 58 | 5.55 | 4.50-6.61 | 5.50 | 4.00-6.80 | 0.949 | 0.873-0.990 | <0.001 |
| DISCERN | ChatGPT vs Copilot | 58 | -3.11 | -4.32--1.90 | -4.10 | -5.20--1.80 | -0.643 | -0.846--0.407 | <0.001 |
| DISCERN | ChatGPT vs Doubao | 58 | 7.28 | 5.93-8.65 | 7.20 | 6.00-8.60 | 0.962 | 0.897-0.997 | <0.001 |
| DISCERN | Gemini vs Copilot | 58 | -8.66 | -9.76--7.64 | -8.30 | -10.20--6.90 | -1.000 | -1.000--1.000 | <0.001 |
| DISCERN | Gemini vs DeepSeek | 58 | -4.90 | -6.06--3.72 | -4.80 | -6.60--3.00 | -0.874 | -0.965--0.739 | <0.001 |
| DISCERN | Gemini vs Doubao | 58 | 1.73 | 0.53-2.93 | 1.50 | -0.80-3.40 | 0.390 | 0.105-0.645 | 0.012 |
| DISCERN | Copilot vs DeepSeek | 58 | 3.76 | 2.54-4.99 | 3.40 | 2.00-5.20 | 0.732 | 0.536-0.880 | <0.001 |
| DISCERN | Copilot vs Doubao | 58 | 10.39 | 9.29-11.53 | 10.00 | 8.40-11.90 | 1.000 | 1.000-1.000 | <0.001 |
| DISCERN | DeepSeek vs Doubao | 58 | 6.63 | 5.41-7.90 | 7.20 | 4.40-9.00 | 0.949 | 0.868-0.990 | <0.001 |
| EQIP | ChatGPT vs Gemini | 58 | 9.24 | 7.45-11.00 | 9.00 | 6.00-11.00 | 0.964 | 0.904-0.995 | <0.001 |
| EQIP | ChatGPT vs Copilot | 58 | -4.66 | -6.34--2.98 | -5.50 | -8.00--3.00 | -0.704 | -0.870--0.494 | <0.001 |
| EQIP | ChatGPT vs DeepSeek | 58 | 2.66 | 0.78-4.40 | 2.00 | 1.00-5.00 | 0.448 | 0.170-0.698 | 0.004 |
| EQIP | ChatGPT vs Doubao | 58 | 13.43 | 11.90-15.12 | 12.50 | 10.00-15.50 | 1.000 | 1.000-1.000 | <0.001 |
| EQIP | Gemini vs Copilot | 58 | -13.90 | -15.67--12.12 | -14.50 | -17.00--11.00 | -0.996 | -1.000--0.982 | <0.001 |
| EQIP | Gemini vs DeepSeek | 58 | -6.59 | -8.43--4.74 | -6.00 | -8.50--4.00 | -0.822 | -0.958--0.646 | <0.001 |
| EQIP | Gemini vs Doubao | 58 | 4.19 | 2.55-5.83 | 5.00 | 1.00-7.00 | 0.651 | 0.423-0.839 | <0.001 |
| EQIP | Copilot vs DeepSeek | 58 | 7.31 | 5.52-9.16 | 7.00 | 4.00-10.00 | 0.857 | 0.709-0.963 | <0.001 |
| EQIP | Copilot vs Doubao | 58 | 18.09 | 16.41-19.72 | 17.00 | 16.00-20.00 | 1.000 | 1.000-1.000 | <0.001 |
| EQIP | DeepSeek vs Doubao | 58 | 10.78 | 9.00-12.69 | 10.50 | 8.50-13.00 | 0.986 | 0.952-1.000 | <0.001 |
| JAMA | ChatGPT vs Gemini | 58 | 0.26 | 0.01-0.50 | 0.00 | 0.00-0.80 | 0.381 | 0.066-0.661 | 0.030 |
| JAMA | ChatGPT vs DeepSeek | 58 | 0.54 | 0.30-0.79 | 0.80 | 0.00-1.00 | 0.648 | 0.373-0.879 | <0.001 |
| JAMA | ChatGPT vs Doubao | 58 | 0.57 | 0.34-0.79 | 0.20 | 0.00-1.00 | 0.728 | 0.494-0.903 | <0.001 |
| JAMA | Gemini vs Copilot | 58 | -0.58 | -0.82--0.35 | -0.80 | -1.00--0.10 | -0.699 | -0.886--0.465 | <0.001 |
| JAMA | Gemini vs DeepSeek | 58 | 0.29 | 0.04-0.53 | 0.40 | 0.00-0.80 | 0.376 | 0.061-0.659 | 0.030 |
| JAMA | Gemini vs Doubao | 58 | 0.31 | 0.09-0.53 | 0.00 | 0.00-0.70 | 0.502 | 0.188-0.764 | 0.007 |
| JAMA | Copilot vs DeepSeek | 58 | 0.87 | 0.66-1.10 | 1.00 | 0.60-1.00 | 0.900 | 0.759-0.986 | <0.001 |
| JAMA | Copilot vs Doubao | 58 | 0.89 | 0.71-1.10 | 1.00 | 0.80-1.00 | 0.949 | 0.863-0.991 | <0.001 |
| GQS | ChatGPT vs Gemini | 58 | 0.48 | 0.28-0.68 | 0.30 | 0.00-1.00 | 0.710 | 0.475-0.885 | <0.001 |
| GQS | ChatGPT vs DeepSeek | 58 | 0.31 | 0.07-0.53 | 0.10 | 0.00-0.80 | 0.436 | 0.120-0.724 | 0.019 |
| GQS | ChatGPT vs Doubao | 58 | 0.47 | 0.25-0.68 | 0.20 | 0.00-0.80 | 0.651 | 0.370-0.879 | <0.001 |
| GQS | Gemini vs Copilot | 58 | -0.61 | -0.81--0.39 | -0.80 | -1.00--0.20 | -0.768 | -0.935--0.543 | <0.001 |
| GQS | Copilot vs DeepSeek | 58 | 0.44 | 0.23-0.66 | 0.50 | 0.10-0.80 | 0.610 | 0.337-0.848 | <0.001 |
| GQS | Copilot vs Doubao | 58 | 0.61 | 0.37-0.85 | 0.90 | 0.80-1.00 | 0.632 | 0.385-0.834 | <0.001 |

Note: Pairwise comparisons were performed using paired Wilcoxon signed-rank tests with Benjamini-Hochberg correction. Paired mean/median differences and rank-biserial correlations are reported with bootstrap 95% CIs. Only significant comparisons are shown.
