# Supplementary material for: Safety, accuracy, empathy, reliability, and readability of large language model chatbot responses to public-facing vegetarian and vegan nutrition advice questions: a cross-sectional comparative study
Source: Front Public Health. 2026 Aug 3;14:1921819. doi: 10.3389/fpubh.2026.1921819 (PMC13478225; doi:10.3389/fpubh.2026.1921819)
Supplement: Supplementary file 14 [file Table_9.docx]

**Supplementary Table S9. Pairwise post hoc comparisons showing statistically significant differences in readability metrics**

| **Metric** | **Comparison** | **N pairs** | **Mean difference** | **Mean difference 95% CI** | **Median difference** | **Median difference 95% CI** | **Rank-biserial correlation** | **Rank-biserial 95% CI** | **Adjusted *P*** |
| --- | --- | --- | --- | --- | --- | --- | --- | --- | --- |
| ARI | ChatGPT vs Gemini | 58 | 2.82 | 2.04-3.58 | 2.90 | 1.99-3.82 | 0.797 | 0.601-0.945 | <0.001 |
| ARI | ChatGPT vs Copilot | 58 | 1.80 | 1.15-2.42 | 1.55 | 0.42-2.53 | 0.686 | 0.475-0.850 | <0.001 |
| ARI | ChatGPT vs DeepSeek | 58 | 1.81 | 0.76-2.74 | 2.26 | 1.15-3.00 | 0.612 | 0.361-0.828 | <0.001 |
| ARI | Gemini vs Copilot | 58 | -1.02 | -1.64--0.41 | -1.39 | -1.94--0.26 | -0.466 | -0.698--0.192 | 0.003 |
| ARI | Gemini vs DeepSeek | 58 | -1.00 | -1.83--0.22 | -0.63 | -1.57-0.07 | -0.323 | -0.583--0.036 | 0.041 |
| ARI | Gemini vs Doubao | 58 | -3.90 | -5.10--2.70 | -4.18 | -5.29--2.90 | -0.769 | -0.930--0.568 | <0.001 |
| ARI | Copilot vs Doubao | 58 | -2.88 | -4.11--1.65 | -3.00 | -4.15--1.91 | -0.603 | -0.815--0.355 | <0.001 |
| ARI | DeepSeek vs Doubao | 58 | -2.90 | -4.24--1.44 | -3.09 | -4.04--2.35 | -0.676 | -0.890--0.433 | <0.001 |
| CL | ChatGPT vs Gemini | 58 | 1.74 | 1.33-2.13 | 2.13 | 1.58-2.33 | 0.834 | 0.669-0.956 | <0.001 |
| CL | ChatGPT vs DeepSeek | 58 | 1.42 | 1.04-1.81 | 1.41 | 1.20-1.87 | 0.826 | 0.668-0.940 | <0.001 |
| CL | Gemini vs Copilot | 58 | -1.43 | -1.82--1.00 | -1.73 | -2.14--1.08 | -0.763 | -0.913--0.564 | <0.001 |
| CL | Gemini vs DeepSeek | 58 | -0.32 | -0.65-0.04 | -0.48 | -0.69--0.05 | -0.344 | -0.611--0.052 | 0.028 |
| CL | Gemini vs Doubao | 58 | -1.91 | -2.47--1.29 | -2.25 | -2.98--1.81 | -0.703 | -0.899--0.462 | <0.001 |
| CL | Copilot vs DeepSeek | 58 | 1.10 | 0.73-1.48 | 1.29 | 0.90-1.48 | 0.716 | 0.509-0.884 | <0.001 |
| CL | Copilot vs Doubao | 58 | -0.49 | -1.04-0.09 | -0.69 | -1.33--0.27 | -0.373 | -0.648--0.075 | 0.019 |
| CL | DeepSeek vs Doubao | 58 | -1.59 | -2.16--0.96 | -1.93 | -2.35--1.60 | -0.660 | -0.883--0.414 | <0.001 |
| FKGL | ChatGPT vs Gemini | 58 | 2.48 | 1.90-3.10 | 2.58 | 1.86-3.29 | 0.841 | 0.675-0.959 | <0.001 |
| FKGL | ChatGPT vs Copilot | 58 | 1.40 | 0.88-1.89 | 1.52 | 0.55-2.02 | 0.675 | 0.454-0.849 | <0.001 |
| FKGL | ChatGPT vs DeepSeek | 58 | 1.52 | 0.69-2.26 | 1.98 | 1.35-2.61 | 0.603 | 0.357-0.820 | <0.001 |
| FKGL | Gemini vs Copilot | 58 | -1.08 | -1.58--0.61 | -1.07 | -1.83--0.51 | -0.576 | -0.785--0.328 | <0.001 |
| FKGL | Gemini vs DeepSeek | 58 | -0.96 | -1.59--0.37 | -0.54 | -1.54--0.14 | -0.411 | -0.661--0.127 | 0.008 |
| FKGL | Gemini vs Doubao | 58 | -3.29 | -4.22--2.33 | -3.29 | -4.52--2.78 | -0.767 | -0.931--0.563 | <0.001 |
| FKGL | Copilot vs Doubao | 58 | -2.21 | -3.22--1.21 | -2.38 | -2.85--1.90 | -0.580 | -0.804--0.335 | <0.001 |
| FKGL | DeepSeek vs Doubao | 58 | -2.33 | -3.39--1.14 | -2.42 | -3.38--1.83 | -0.667 | -0.888--0.403 | <0.001 |
| GFI | ChatGPT vs Gemini | 58 | 2.83 | 2.13-3.52 | 2.72 | 2.05-3.63 | 0.861 | 0.707-0.970 | <0.001 |
| GFI | ChatGPT vs Copilot | 58 | 1.30 | 0.75-1.85 | 1.27 | 0.67-1.99 | 0.590 | 0.351-0.790 | <0.001 |
| GFI | ChatGPT vs DeepSeek | 58 | 1.74 | 0.79-2.59 | 2.15 | 1.11-3.09 | 0.613 | 0.371-0.808 | <0.001 |
| GFI | Gemini vs Copilot | 58 | -1.54 | -2.07--1.00 | -1.55 | -2.25--1.09 | -0.684 | -0.866--0.458 | <0.001 |
| GFI | Gemini vs DeepSeek | 58 | -1.09 | -1.77--0.47 | -0.76 | -1.85--0.25 | -0.440 | -0.674--0.161 | 0.004 |
| GFI | Gemini vs Doubao | 58 | -3.50 | -4.54--2.41 | -3.67 | -5.12--2.65 | -0.748 | -0.927--0.531 | <0.001 |
| GFI | Copilot vs Doubao | 58 | -1.96 | -3.01--0.81 | -1.96 | -3.14--1.30 | -0.510 | -0.749--0.260 | 0.001 |
| GFI | DeepSeek vs Doubao | 58 | -2.40 | -3.58--1.09 | -2.56 | -3.76--1.84 | -0.650 | -0.871--0.406 | <0.001 |
| SMOG | ChatGPT vs Gemini | 58 | 1.96 | 1.47-2.44 | 1.88 | 1.43-2.59 | 0.853 | 0.697-0.961 | <0.001 |
| SMOG | ChatGPT vs Copilot | 58 | 0.99 | 0.62-1.37 | 0.93 | 0.50-1.58 | 0.635 | 0.401-0.823 | <0.001 |
| SMOG | ChatGPT vs DeepSeek | 58 | 1.21 | 0.60-1.78 | 1.55 | 0.78-1.95 | 0.611 | 0.373-0.812 | <0.001 |
| SMOG | Gemini vs Copilot | 58 | -0.97 | -1.37--0.59 | -0.97 | -1.48--0.62 | -0.643 | -0.840--0.399 | <0.001 |
| SMOG | Gemini vs DeepSeek | 58 | -0.75 | -1.19--0.29 | -0.51 | -1.25--0.23 | -0.424 | -0.670--0.152 | 0.006 |
| SMOG | Gemini vs Doubao | 58 | -2.38 | -3.07--1.64 | -2.78 | -3.44--1.99 | -0.750 | -0.915--0.545 | <0.001 |
| SMOG | Copilot vs Doubao | 58 | -1.41 | -2.14--0.60 | -1.52 | -2.24--1.10 | -0.522 | -0.765--0.248 | <0.001 |
| SMOG | DeepSeek vs Doubao | 58 | -1.63 | -2.42--0.74 | -1.74 | -2.38--1.34 | -0.647 | -0.870--0.384 | <0.001 |
| FRES | ChatGPT vs Gemini | 58 | -11.86 | -14.31--9.25 | -12.98 | -14.18--10.17 | -0.877 | -0.977--0.737 | <0.001 |
| FRES | ChatGPT vs Copilot | 58 | -3.52 | -5.49--1.59 | -5.00 | -6.64--0.64 | -0.473 | -0.704--0.196 | 0.002 |
| FRES | ChatGPT vs DeepSeek | 58 | -7.98 | -10.77--5.18 | -8.94 | -11.74--6.23 | -0.742 | -0.903--0.535 | <0.001 |
| FRES | Gemini vs Copilot | 58 | 8.34 | 6.02-10.64 | 9.82 | 6.56-12.72 | 0.786 | 0.615-0.925 | <0.001 |
| FRES | Gemini vs DeepSeek | 58 | 3.87 | 1.69-6.07 | 3.66 | 1.19-5.41 | 0.475 | 0.217-0.716 | 0.002 |
| FRES | Gemini vs Doubao | 58 | 14.11 | 10.12-17.96 | 15.69 | 12.90-17.99 | 0.758 | 0.548-0.939 | <0.001 |
| FRES | Copilot vs DeepSeek | 58 | -4.46 | -6.93--1.91 | -5.48 | -7.98--2.20 | -0.510 | -0.738--0.259 | 0.001 |
| FRES | Copilot vs Doubao | 58 | 5.77 | 1.79-9.58 | 7.50 | 3.58-11.43 | 0.495 | 0.214-0.750 | 0.002 |
| FRES | DeepSeek vs Doubao | 58 | 10.23 | 5.72-14.22 | 10.50 | 8.96-14.13 | 0.696 | 0.452-0.909 | <0.001 |

Note: Pairwise comparisons were performed using paired Wilcoxon signed-rank tests with Benjamini-Hochberg correction. Paired mean/median differences and rank-biserial correlations are reported with bootstrap 95% CIs. Only significant comparisons are shown.
